# Genome-Wide Identification and Evaluation of Reference Genes for Quantitative RT-PCR Analysis during Tomato Fruit Development

**DOI:** 10.3389/fpls.2017.01440

**Published:** 2017-08-29

**Authors:** Yuan Cheng, Wuying Bian, Xin Pang, Jiahong Yu, Golam J. Ahammed, Guozhi Zhou, Rongqing Wang, Meiying Ruan, Zhimiao Li, Qingjing Ye, Zhuping Yao, Yuejian Yang, Hongjian Wan

**Affiliations:** ^1^State Key Laboratory Breeding Base for Zhejiang Sustainable Pest and Disease Control, Institute of Vegetables, Zhejiang Academy of Agricultural Sciences Hangzhou, China; ^2^Zhejiang Agricultural Technology Extension Center Hangzhou, China; ^3^Suzhou Polytechnic Institute of Agriculture Suzhou, China; ^4^Department of Horticulture, Zhejiang University Hangzhou, China

**Keywords:** qPCR analysis, normalization, reference gene (RG), tomato, fruit development

## Abstract

Gene expression analysis in tomato fruit has drawn increasing attention nowadays. Quantitative real-time PCR (qPCR) is a routine technique for gene expression analysis. In qPCR operation, reliability of results largely depends on the choice of appropriate reference genes (RGs). Although tomato is a model for fruit biology study, few RGs for qPCR analysis in tomato fruit had yet been developed. In this study, we initially identified 38 most stably expressed genes based on tomato transcriptome data set, and their expression stabilities were further determined in a set of tomato fruit samples of four different fruit developmental stages (Immature, mature green, breaker, mature red) using qPCR analysis. Two statistical algorithms, geNorm and Normfinder, concordantly determined the superiority of these identified putative RGs. Notably, SlFRG05 (Solyc01g104170), SlFRG12 (Solyc04g009770), SlFRG16 (Solyc10g081190), SlFRG27 (Solyc06g007510), and SlFRG37 (Solyc11g005330) were proved to be suitable RGs for tomato fruit development study. Further analysis using geNorm indicate that the combined use of SlFRG03 (Solyc02g063070) and SlFRG27 would provide more reliable normalization results in qPCR experiments. The identified RGs in this study will be beneficial for future qPCR analysis of tomato fruit developmental study, as well as for the potential identification of optimal normalization controls in other plant species.

## Introduction

Tomato (*Solanum lycopersicum*) is an economically important horticultural crop in terms of production, flavor, and nutritional value of fruits. During the course of development and ripening, tomato fruits undergo a number of physiological and biochemical processes that bring forth the overall changes in fruit size, color, texture, and aroma (Klee and Giovannoni, 2011; Ruiz-May and Rose, 2012). Moreover, tomato is considered as an important model for genetic and molecular studies, partly due to its typical climacteric fruit property (Colombiet et al., 2016). A number of studies had been carried out to improve agronomic traits of tomato fruits, including size, pigment content, and flavor substances focusing on the metabolic and regulatory networks (Klee and Giovannoni, 2011; Ruiz-May and Rose, 2012; Tieman et al., 2017). Recent developments in genomic resources and bioinformatics tools (e.g., Genome-wide association study, GWAS) have enabled rapid elucidation of the complicated biological processes that occur during fruit development. Moreover, relative gene expression profiles during fruit development provide valuable clues for understanding the biological functions of the corresponding genes. So far, quantitative real-time PCR (qPCR) is considered as one of the most efficient tools for the measurement of transcript abundance of a gene due to its high accuracy, sensitivity, and reproducibility (Ginzinger, 2002; Bustin and Nolan, 2004; Gachon et al., 2004; Bustin et al., 2005).

In qPCR experiments, the reliability of the results predominantly depends on the appropriateness of RGs used for normalization, which should be stably expressed under the given experimental conditions (Pfaffl, 2004; Huggett et al., 2005). Highly stable expression of RGs could effectively remove non-biological variations including the difference in amounts, variability in enzymatic efficiency of reverse transcriptase, and sample differences in the overall transcriptional activity (Suzuki et al., 2000; Bustin et al., 2005; Huggett et al., 2005; Exposito-Rodriguez et al., 2008; Gutierrez et al., 2008). Generally speaking, an ideal RG should be a gene that is stably expressed under various experimental conditions or among different tissues (Czechowski et al., 2005; Huggett et al., 2005; Exposito-Rodriguez et al., 2008; Dekkers et al., 2012; Wang et al., 2012). Housekeeping genes (HKGs) encoding, e.g., GAPDH, Actin, UBI, and 18 sRNAs, are usually regarded as suitable normalization controls (Stürzenbaum and Kille, 2001). However, some previous studies reported that the transcription of several HKGs can be fluctuated considerably under certain conditions (Czechowski et al., 2005; Jain et al., 2006; Gutierrez et al., 2008; Jian et al., 2008; Jarosova and Kundu, 2010), which illustrates the importance of systematic identification or validation of optimal RGs in order to avoid inaccurate results (Gutierrez et al., 2008; Guenin et al., 2009). In practice, the expression levels of most RGs are proved to be dependent on the specific conditions, including experimental treatments, tissue types, or developmental stages (Czechowski et al., 2005; Jian et al., 2008; Jarosova and Kundu, 2010). Hence, no single RG is widely applicable for different experimental conditions. Systematic evaluation of RGs must be conducted on each qPCR experiment before their use (Bustin et al., 2009; Jacob et al., 2013). Furthermore, it has been well-recognized that in some cases, one single RG may not be adequate for reliable normalization in gene expression analysis (Yoo et al., 2009; Cassan-Wang et al., 2012). To date, some common statistical algorithms, including geNorm (Vandesompele et al., 2002), NormFinder (Andersen et al., 2004), and Bestkeeper (Pfaffl, 2004), have been developed to determine the expression stabilities of RGs, which effectively simplify the selection of appropriate RGs for qPCR analysis.

Over the decades, a good number of stably expressed RGs have specifically been identified for normalization in the fruits of several fruit crops, such as papaya (Zhu et al., 2012), blueberry (Die and Rowland, 2013), and watermelon (Kong et al., 2015). For tomato, although suitable RGs have been identified under different experimental conditions including biotic/abiotic stresses (Løvdal and Lillo, 2009; Mascia et al., 2010) and various tissues of different developmental stages (Suzuki et al., 2000; Dekkers et al., 2012), very limited number of RGs in tomato fruit have been characterized so far (Coker and Davies, 2003; Baldassarre et al., 2015). Moreover, we noticed that most studies involving RG identification, including those relevant to tomato fruit developmental studies, were mainly based on the evaluation or validation of some already known candidate RGs (Mostly HKGs), which are convenient for implementation but also greatly limit the choice of best RGs. With the availability of tomato genome sequence and subsequent transcriptome data (SGN:Sol genomics network, https://solgenomics.net/; TFGD: Tomato functional genomics database,http://ted.bti.cornell.edu/), our study was aimed to identify some novel RGs for qPCR analysis of tomato fruit development within the entire genome level.

In this study, we initially evaluated the expression stabilities of all the tomato (*S. lycopersicum* L.) genes during various fruit developmental stages based on the RNA-seq data. A total of 38 novel genes stably expressed were identified as putative RGs and were further evaluated through qPCR analysis. Moreover, using two different statistical algorithms (geNorm and Normfinder), five optimal RGs were identified as optimal RGs for normalization during different stages of tomato fruit development. Furthermore, we also found that the combined use of two top-ranked RGs (*SlFRG03* and *SlFRG27*) would potentially improve the accuracy of the qPCR results. Thus, based on the analysis of the entire tomato genome database, we comprehensively identified and evaluated the optimal RGs through large-scale biological information mining and qPCR techniques. These results not only provide useful RG resources for accurate gene expression studies in tomato fruit, but also shed light on the RG identification in fruit developmental study of other plant species.

## Materials and methods

### Collection and evaluation of the previously reported RGs

**Figure 1:**
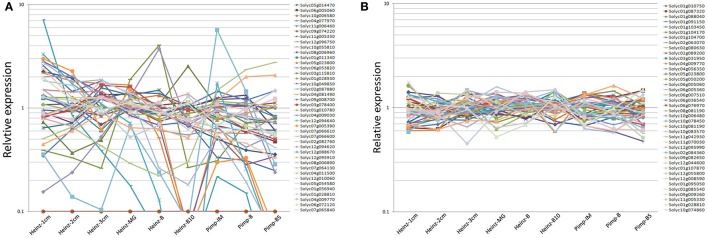
Relative expression analysis of previously reported RGs **(A)** and newly identified RGs **(B)** over different tomato fruit developmental stages. Expression levels (RPKM values) were derived from RNA seq dataset (http://ted.bti.cornell.edu/). Relative expression level per gene were calculated by dividing the expression value (RPKM value) by the average expression level across various developmental stages of tomato fruits (Heinz-1 cm, Heinz-2 cm, Heinz-3 cm, Heinz-MG, Heinz-B, Heinz-MG, Heinz-B10, Pimp-IM, Pimp-B, and Pimp-B5) (Supplementary Tables 1–3).

Firstly, the potential tomato RGs reported in previous studies (Coker and Davies, 2003; Exposito-Rodriguez et al., 2008; Løvdal and Lillo, 2009; Mascia et al., 2010; Müller et al., 2015) were selected to evaluate the expression stabilities during different stages of tomato (*S. lycopersicum* L.) fruit development based on the RNA-seq data downloaded from the TFGD (http://ted.bti.cornell.edu/). Furthermore, the orthologous genes of 11 potential RGs for watermelon fruit development (Kong et al., 2015) were identified. All the potential RGs selected from RNA-seq data were evaluated for expression stability. Details including accession number, gene locus, gene description, and RNA-seq values were listed in Table 1 and Supplementary Table 1. The corresponding gene sequences of these candidate RGs were collected from NCBI (National Center for Biotechnology Information: https://www.ncbi.nlm.nih.gov/), SGN (https://solgenomics.net/), TFGD (http://ted.bti.cornell.edu/) (Fraser et al., 1994), and CuGenDB (http://www.icugi.org/). Through Blastn search, the orthologous genes (E-value was set at 1e^−5^) were collected in tomato genome databases (SGN) (Table 1). The Reads Per Kilobase Million (RPKM) value of each reported RG was collected from RNA-seq data, and the average expression (AVE) and standard deviation (*SD*) values in different fruit developmental stages of fruit were calculated (Supplementary Table 1; Table 1). Relative expression level per gene was calculated through dividing the expression value of each fruit development stage by AVE (Figure 1A). CV (co-efficient variation) value of each gene during fruit development was calculated as the ratio of the *SD* to the AVE (Supplementary Table 3).

**Table 1:** Previously reported RGs in tomato or watermelon.

**Gene symbol**	**Accession number**	**Gene locus**	**Gene description**	**Sequence identities (%)**
GAPDH	U97257	Solyc05g014470	Glyceraldehyde 3-phosphate dehydrogenase	99
EF1α	X53043	Solyc06g005060	Elongation factor 1-alpha	98
RPL8	X64562	Solyc10g006580	Ribosomal protein L2	99
DNAJ	AF124139	Solyc11g006460	DNAJ-like protein	96
TUA	AC122540	Solyc09g074220	Musmusculus BAC clone	99
Actin	Q96483	Solyc11g005330	Actin-51	99
TAPG4	U70481.1	Solyc12g096750	Abscission polygalacturonase	99
CHI9	NM001247474	Solyc10g055810	Chitinase (CHI9)	99
ACT	BT013707	Solyc03g0'78400	Actin	100
CYP	AK326854	Solyc01g108340	Cyclophilin Elongation factor 1-α	100
GAPDH	U93208	Solyc03g111010	Glyceraldehyde 3-phosphate dehydrogenase	98
UBI	X58253	Solyc01g056940	Ubiquitin 3	99
UK	AK322232	Solyc01g088480	Uridylate kinase	100
18S rRNA	X51576	Solyc11g051210	18S rRNA	97
TBP	SGN-U329249	**Solyc01g028930**	TATA binding protein 2	100
TIP41	SGN-U321250	**Solyc10g049850**	TIP41-like family protein	99
SAND	SGN-U316474	**Solyc03g115810**	SAND family protein	99
CAC	SGN-U314153	Solyc08g006960	Clathrin adaptor complexes medium subuit	100
Expressed	SGN-U346908	Solyc07g025390	Expressed sequence	98
PK	TC123837	Solyc07g066610	Phosphoglycerate kinase	100
CBCP	TC115713	Solyc01g028810	Chaperonin-60 beta chain prec	100
DNAJ	TC123959	Solyc04g009770	DNAJ-like protein	100
AT5G	TC123964	Solyc06g072120	AT5g	100
ClCAC	Cla016178	Solyc06g061150	Clathrin adaptor complex subunit	95
ClPP2A	Cla021905	Solyc01g011340	Protein phosphatase 2A regulatory subunit A	82
ClRAN	Cla012277	Solyc05g023800	A member of RAN GTPase gene family	82
ClRPS15	Cla021565	Solyc06g053820	Cytosolic ribosomal protein S15	84
ClSAND	Cla001870	**Solyc03g115810**	SAND family protein	80
ClTBP2	Cla011119	**Solyc01g028930**	TATA binding protein 2	82
ClTIP41	Cla016074	**Solyc10g049850**	TIP41-like family protein	86
ClTUA5	Cla003129	Solyc02g087880	Alpha tubulin 5	80
ClTUB	Cla022418	Solyc04g081490	β-tubulin	84
ClUPL7	Cla017746	Solyc09g008700	Ubiquitin-protein ligase 7	80
Cl18SrRNA	Cla010159	Solyc05g007050	18SrRNA	82
		Solyc10g006100	Transcription initiation factor TFIID subunit 6	100
		Solyc07g062920	Genomic DNA chromosome 5 TAC clone K19P17	100
		Solyc01g111780	Importin beta-2 subunit	100
		Solyc06g051420	PHD finger family protein	100
		Solyc12g057120	Subunit VIb of cytochrome c oxidase	100
		Solyc01g009290	Polyribonucleotide 5'-hydroxyl-kinase Clp1	100
		Solyc09g018730	Ubiquitin carboxyl-terminal hydrolase family 1	100
		Solyc02g088110	Polypyrimidine tract-binding protein-like	100
		Solyc08g060860	P1 clone MSJ11	100
		Solyc09g009640	U6 snRNA-associated Sm-like protein LSm7	100
		Solyc04g015370	Acyl carrier protein	100
		Solyc08g005140	Serine/threonine-protein kinase BUD32	100
		Solyc02g062920	Splicing factor U2AF large subunit	100
		Solyc10g076910	ATP-dependent RNA helicase-like protein	100
		Solyc03g121980	Developmentally-regulated GTP-binding protein 2	100
		Solyc01g097140	Dual-specificity tyrosine-phosphatase CDC25	100
		Solyc07g007040	Zinc finger CCCH-type protein	100
		Solyc06g069310	Nuclear transcription factor Y subunit B-6	100
		Solyc03g078020	Peptide chain release factor 1	100
		Solyc10g078180	cyclin gene family	100
		Solyc02g089230	DSBA oxidoreductase family protein	100
		Solyc06g036720	HLA-B associated transcript 3	100
		Solyc01g109620	Unkown	100
		Solyc07g064510	Polyadenylate-binding protein 2	100
		Solyc11g071930	DnaJ homolog subfamily C member 8	100
		Solyc06g084000	Heterogeneous nuclear ribonucleoprotein K	100
		Solyc04g009230	Mitosis protein dim1	100
		Solyc06g073870	DNA-directed RNA polymerase II subunit RPB4	100
		Solyc09g055760	T-snare	100
		Solyc12g005780	TraB family protein	100
		Solyc04g008610	Histone acetyltransferase	100
		Solyc04g015300	Alpha/beta hydrolase	100
		Solyc10g005800	CWC15 homolog	100
		Solyc12g021130	3-beta-hydroxysteroid dehydrogenase-like	100
		Solyc01g079330	RNA helicase DEAD3	100
		Solyc07g041550	RNA polymerase-associated protein Ctr9 homolog	100
		Solyc03g059420	Sister chromatid cohesion 2	100
		Solyc11g071950	Unknown	100
		Solyc12g099570	Heat shock factor binding protein 2	100
		Solyc10g044900	CASTOR protein	100
		Solyc10g084270	Importin alpha-2 subunit	100
		Solyc06g016750	Transcription factor	100
		Solyc02g092380	Peptidyl-prolylcis-trans isomerase	100
		Solyc05g052960	BTB/POZ domain containing protein	100
		Solyc06g009860	Mercaptopyruvate sulfurtransferase-like protein	100
		Solyc10g008950	Guanylate-binding protein 10	100
		Solyc10g055450	Ubiquitin-protein ligase 4	100
		Solyc05g006580	Unknown	100
		Solyc03g121310	RWD domain-containing protein	100
		Solyc09g010180	Unknwon	100

*Detailed information was derived from NCBI (https://www.ncbi.nlm.nih.gov/), TFGD (https://www.ncbi.nlm.nih.gov/), CuGenDB (http://www.icugi.org/), and SGN (https://solgenomics.net/). The three redundant genes were labeled in bold*.

### Identification of stably expressed genes through mining fruit RNA-seq data set

Through mining the entire set of RNA-seq data file (TFGD), genes with medium expression (200 < RPKM value < 2,000) at all stages of fruit development were selected (Supplementary Table 2). The CV value of each gene was calculated (Supplementary Table 2) and the genes with CV < 0.35 were chosen as putative RGs in the following qPCR analysis (Table 2; Supplementary Table 3).

**Table 2:** Description of the newly identified RGs, primer sequences, and amplification characteristics.

**Gene ID**	**Gene locus^a^**	**Gene description**	**Primer sequence (5′–3′)**	**Product size (bp)**	**E**	***R*^2^**
*SlFRG01*	Solyc06g005360	Actin depolymerizing factor 3	F: TGACAAAGGAAAGTGTCCCA	133	0.76	0.994
			R: ATCTGAATTCCGTCCAGCTC			
*SlFRG02*	Solyc01g088040	Unknown	F: GTTGATGAAGGGAGAGCCAT	95	0.78	0.998
			R: GAGAGTAGATCACGGCAGCA			
*SlFRG03*	Solyc02g063070	Unknown	F: GGCTGAACTGGCTCCTACTC	109	0.77	0.999
			R: TTTCGCAAGGTTACAAGCAC			
*SlFRG04*	Solyc12g095990	RNA helicase DEAD40	F: ACTCACGGAGACATGGATCA	107	0.78	0.999
			R: TCAATACCACGAGCAAGGAG			
*SlFRG05*	Solyc01g104170	Ankyrin repeat domain-containing protein	F: CAAAGGTTGATGCATTGGAC	110	1.05	0.958
			R: AGTTACAGCGGCTCCATTCT			
*SlFRG06*	Solyc11g070030	Unknown	F: CCACACGACACTTCCATCTC	111	0.90	1
			R: TCAGCCGGTTTAGATATCCC			
*SlFRG07*	Solyc01g091150	Golgi SNAP receptor complex member	F: CAATCAAGAAGGATCCAGCA	149		
			R: GCCTTGCCAGTTTCTGTGTA			
*SlFRG08*	Solyc11g042930	E3 ubiquitin ligase	F: CTGGCTGCCAACTACTTGAA	71	0.82	0.983
			R: TTAATCATGTCTGCCACGGT			
*SlFRG09*	Solyc03g031950	unknown	F: CCCAATTTCTTCCTCCGTAA	108	0.98	0.954
			R: TGGCCTAAGAAGTCCGAATC			
*SlFRG10*	Solyc10g078450	U6 snRNA-associated Sm-like protein	F: TATAGCAATGGAGCAAACCG	112	0.75	0.997
			R: TGTCCTCTTTGATGTGCTGA			
*SlFRG11*	Solyc05g023800	Uncharacterized protein	F: ACAGGCACTGCATGAAGAAG	109	0.91	0.993
			R: GTGAAACTGATCCGCTCTGA			
*SlFRG12*	Solyc04g009770	Ran protein/TC4 protein 1	F: AATCAAGTCCCAACCTGGAG	93	0.90	0.989
			R: TTCATGAATGGCCTTTGGTA			
*SlFRG13*	Solyc01g103450	DNAJ chaperone	F: AATGACAAGGTTTCCAAGGC	100		
			R: CTTCTAATCCAGCAATGCGA			
*SlFRG14*	Solyc06g076970	Peptidyl-prolylcis-trans isomerase	F: CTCTGCAGTTTGGTCGTGAT	126	0.84	0.983
			R: AATACGACCGGCAGGTTTAC			
*SlFRG15*	Solyc01g010750	Stress responsive protein	F: GGATGAAGAGGGATTTCCAA	120	0.96	0.995
			R: TCAAGAACGTCGGGATTGTA			
*SlFRG16*	Solyc10g081190	Leucine-rich repeat receptor-like kinase	F: AACAGGACCAATCCCAAGAG	147	0.99	0.995
			R: TCAAGTCGAGGATTGTGCTC			
*SlFRG17*	Solyc04g056350	Zinc finger family protein	F: ATGCAGCAACGACTAACAGG	99	0.84	0.993
			R: TGCAAAGAGGACATTCAAGC			
*SlFRG18*	Solyc01g104700	Ran protein/TC4 protein ran2b	F: ACTTAGCTGCACAGGCATTG	125	0.91	0.989
			R: ATGGAAAGGTGCAACAGTGA			
*SlFRG19*	Solyc10g006480	Ubiquitin	F: CAACCCTTCACTTGGTCCTT	91	0.86	0.988
			R: CTTTCCACCTCAAGGGTGAT			
*SlFRG20*	Solyc10g083570	Fructose-bisphosphatealdolase	F: TGTTGAGCCTGAGATCCTTG	136	1.01	0.992
			R: GGGCTTCAACAATGTACCCT			
*SlFRG21*	Solyc02g089200	SEP subfamily of the MADS-box gene f	F: GGTGGTGAGCAAAGTCTCAA	67	1.10	0.988
			R: GAGGTTGGAAGAAACCCTGA			
*SlFRG22*	Solyc08g081190	plasma membrane intrinsic protein	F: GACCACTGGATCTTCTGGGT	99	1.39	0.999
			R: TTAAGACCTGTGGAATGGCA			
*SlFRG23*	Solyc06g036540	Unknown	F: GGGCTTATCAAGGAAGGGAT	138	0.86	0.992
			R: GGCACCTCTTGTCACCTTT			
*SlFRG24*	Solyc02g080630	Lactoylglutathionelyase	F: GGATGCAAGTGGTGAAGAAA	144	1.03	0.996
			R: CGTATGCATTTCCCTTTGTG			
*SlFRG25*	Solyc05g050200	Eukaryotic translation initiation factor 1A	F: CGAAGAACAAGGGTAAGGGA	137	0.90	0.993
			R: ACATCGTCCATTACCAAGCA			
*SlFRG26*	Solyc01g087320	tRNA-dihydrouridine synthase A	F: TGTTGCTCCCATGATGAAAT	97	0.94	0.994
			R: AGCAGCAAGCATTTCTGTGT			
*SlFRG27*	Solyc06g007510	Ubiquitin-conjugating enzyme E2	F: GCTCTCTGTTGACAGACCCA	108	1.10	0.998
			R: GAGTCCAGCTACGAGCAGTG			
*SlFRG28*	Solyc06g005060	elongation factor 1-alpha	F: TTGGTGGTATTGACAAGCGT	124	0.84	0.991
			R: GTGATACCACGCTCACGTTC			
*SlFRG29*	Solyc02g084360	V-type proton ATPase 16 kDaproteo lipid	F: TGCGCCTTTCTTCGGTTTCC	88	0.94	0.993
			R: CACACCGCTCTTCGCTGTTC			
*SlFRG30*	Solyc09g082650	Acireductonedioxygenase	F:GGCAAGAATATGTTGAGACGTTTGTCA	147	0.80	0.983
			R:CATCAAGAACAGCAAAGCCACCA			
*SlFRG31*	Solyc12g044600	NADP-malic enzyme	F: TCAGCGCATATTGCTGCCAA	125	0.90	0.996
			R: CTGCGGTATGCTGGGCTGTA			
*SlFRG32*	Solyc01g107870	Poly(A) RNA binding protein	F: ACCTGGAATGAGGCCTGGTG	150	0.91	0.99
			R: TGTTGCTGCATCAGCGGAAC			
*SlFRG33*	Solyc12g055800	Unknown	F: GCTTCAGACTTGGCCTGTACG	127	0.91	1
			R: GGCACAAGTTCCACCAAGCA			
*SlFRG34*	Solyc12g008590	Acireductonedioxygenase	F: TCGACGATCACCTCCTGTGTG	81	1.12	0.962
			R: CCCAAACAGCGCCGTCAAG			
*SlFRG35*	Solyc02g085540	NADH ubiquinone oxidoreductase	For: CGCCTGGACAGTCCCGTAAA	127	0.87	0.998
			R: TGGGCCCAAGTTTCAAGGGT			
*SlFRG36*	Solyc09g009260	fructose-1,6-bisphosphate aldolase	F: CTGAGTACACCGTCCGTGCT	102	0.90	0.996
			R: GGTTGACGGTGGCTTCCTCT			
*SlFRG37*	Solyc11g005330	Actin	F: GGAGATTGAAACTGCCAGGAGCA	143	1.00	0.915
			R: CTGCAGCTTCCATACCAATCATGG			
*SlFRG38*	Solyc01g028810	chaperonin	F:GTTGTGTGGTGGTTGAGATCAAGG	74	0.99	0.915
			R:CCGTATCCTGAGTTGTCCATCGG			
	Solyc01g095050	Negatively light-regulated protein	No primer			
	Solyc10g074860	Unknown	No primer			

a *Tomato gene ID in SGN (https://solgenomics.net/). E: Amplification efficiency; R^2^: Correlation coefficient*.

### Plant materials

The tomato (*S. lycopersicum* L.) inbred line “S14” was used in the current experiments. Generally, the fruits of this genotype become completely mature (red flash) 45 days after pollination. On month old tomato seedlings were transplanted to the greenhouse at Zhejiang Academy of Agricultural Sciences, Hangzhou, China (east longitude 120°2″, north latitude 30°27″) on August 5th (late summer), 2016. Field management was implemented following the standard commercial practices. Tomato fruits were harvested at four developmental stages (Immature: IM, Mature Green: MG, Breaker: B, and Mature Red: MR) from October to November (autumn), 2016. Three fruits were randomly collected at each sampling point, with each of them representing a biological replicate. All the samples were frozen in liquid nitrogen and stored at –80°C for subsequent experiments.

### Total RNA isolation and cDNA synthesis

Total RNA from collected samples was isolated using TRIZOL reagent according to the manufacturer's protocol (Tiangen, Beijing, China) as described previously(Cheng et al., 2016). The concentration and purity of extracted RNA were measured using a BioDropULite spectrophotometer (Biochrom, England), and RNA samples with A260/A280>1.8 and A260/A230>2.0 (indicating good RNA quality) were used for following experiments. All RNA samples were adjusted to the same concentration in order to ensure that the RNA input was homogenized for subsequent reverse transcription reactions using a mix of random primers. Then, according to the manufacturer's instruction (TIANGEN, Beijing, China), genomic DNAs (gDNA) were eliminated from the RNA samples and single-stranded cDNAs were synthesized.

### Primer design and qPCR analysis

Gene-specific primers were designed using Real-time PCR (TaqMan) primer design (https://www.genscript.com/) as listed in Table 2. The qPCR experiments was performed in a 96-well plate using SYBR Green-based PCR assay. A 20 μL reaction mixture [6 μL diluted cDNA (10 ng), 10 μL SYBR Green PCR Master Mix (Invitrogen, USA), 250 nM of each primer and, 0.1 μL ROX] was subjected to the following procedure: 10 min at 94°C, 30 cycles of 45 s at 94°C, 45 s at 55°C, and 1 min at 72°C following a 7 min extension at 72°C (ABI real time PCR system, USA). Three technical duplications performed for all of the RGs. The amplified products were resolved by 1.5% agarose gel electrophoresis (Supplementary Figure 1). The melting curves were created and exhibited for all the investigated qPCR products in the qPCR experiments (Supplementary Figure 2).

### Evaluation of RG expression stability using geNorm and normfinder

The expression levels of the detected genes were obtained through the qPCR analysis and the results were demonstrated as Ct values (Supplementary Table 4). The amplification efficiency (E) and correlation coefficient (*R*^2^) for each gene were calculated using the standard curve method by amplifying the 10-fold serial dilution of cDNA samples. The amplification efficiency (E) was calculated with the formula: E = (10^−1/slope^−1). The geNorm and NormFinder software packages were used to evaluate the gene expression stability as described before in this study (Vandesompele et al., 2002; Andersen et al., 2004). The geNorm applet not only provides a measure of gene expression stability value (M), but also creates pairwise variation values (V) to determine the minimum number of RGs required for reliable normalization, no additional genes were required when the pairwise variation (Vn/n+1) was lower than 0.15 (Vandesompele et al., 2002). The NormFinder measured the variations across groups and determine the expression stability of each tested gene (Andersen et al., 2004). Lower stability values (M) in both geNorm and Normfinder implied the higher expression stability of the genes. The stability values (M) obtained from geNorm and NormFinder were listed in Supplementary Table 5.

## Results

### Evaluation of previously reported RGs during tomato fruit development

In our study, tomato (*S. lycopersicum* L.) genome database in the SGN (https://solgenomics.net/) and transcriptome data derived from the TFGD (http://ted.bti.cornell.edu/) were used for analysis. Based on the previously reported RGs in tomato (Coker and Davies, 2003; Exposito-Rodriguez et al., 2008; Løvdal and Lillo, 2009; Mascia et al., 2010; Müller et al., 2015), 73 reported potential RGs of tomato were identified (Table 1). Moreover, by collecting the cDNA sequences of 11 previously reported candidate RGs for watermelon fruit study (From CuGenDB: Cucurbit genomics database, http://www.icugi.org/) (Kong et al., 2015), we subsequently collected their corresponding orthologs in tomato through blastN in the SGN (Table 1). Thus, a total of 84 tomato potential RGs were collected in the present study. Among all the reported RGs and their corresponding orthologs, three genes (Solyc01g028930, Solyc10g049850, and Solyc03g115810) were found to be redundant. Thus, a total of 81 previously reported RGs were collected and listed in Table 1.

According to the RNA-seq data derived from TFGD, RPKM values of the 81 previously reported RGs in five different fruit developmental stages (1, 2, 3 cm, MG [Mature green], B [Breaker], B10 [10 days after breaker]) of accession “Heinz” and in three developmental stages (IM [Immature], B [Breaker], B5 [5 days after breaker]) of accession “Pimp” were used to evaluate their expression stabilities (Supplementary Table 1). The CV values were calculated as described in Materials and Methods (Supplementary Table 3). Based on the RPKM values shown in Supplementary Table 1, we analyzed the relative expression of the 81 reported RGs among different developmental stages of tomato fruit. As shown in Supplementary Table 3, nearly 30% (24/81) of the 81 reported RGs demonstrated high expression variations (CV>0.35) during tomato fruit development (Figure 1A; Supplementary Table 3). Some CV values were even higher than 2.0 (Solyc12g096750/Solyc06g009860 [3.00], Solyc02g087880 [2.31]), suggesting their poor expression stabilities during tomato fruit development. Further analysis demonstrated that although the remaining 57 stably expressed reported RGs have CV values lower than 0.35, most of them (52/57) had the average expression levels (RPKM values) lower than 200, and some RPKM values of them (Solyc09g074220, Solyc12g096750, Solyc11g051210, and Solyc06g009860) were even close to 0 (low transcription level or undetected signal) (Supplementary Table 3), which indicated that these 52 reported RGs may not be qualified for normalization due to their low expression levels. According to Supplementary Table 3, only the PPKM values of five previously reported RGs, Solyc06g005060 (1088.0), Solyc11g005330 (581.1), Solyc01g028810 (509.5), Solyc04g009770 (357.6), and Solyc05g023800 (359.9), were high enough (>200) to be considered as candidate RGs in tomato fruit. Hence, we came to the conclusion that most of the previously reported candidate RGs identified so far were not well-qualified for normalization during tomato fruit development.

### Identification of putative RGs based on RNA-seq data mining

To comprehensively identify qualified RGs for tomato fruit, we searched the entire set of RNA-seq data derived from TFGD, and a total of 56 genes with RPKM values ranged between 200 and 2,000 among all developmental stages of tomato fruit were identified, by searching the derived RNA-seq data (Supplementary Table 2). The CV values of these genes were calculated, and more than 70% (40/56) of them were shown to be lower than 0.35 (Supplementary Table 3). The 40 genes were listed in details in Table 2. Further analysis revealed that the genes identified from the entire genome level (Figure 1B) were generally more stably expressed than those previously reported as candidate RGs (Figure 1A) during tomato fruit development.

### qPCR analysis of the putative RGs in tomato fruit development process

Next, we intended to validate the expression stabilities of the selected 40 putative RGs by qPCR analysis. When designing the primers using genescript online tool (https://www.genscript.com/), we found that proper primers of two candidate RGs (Solyc01g095050 [321 bp] and Solyc10g074860 [72 bp]) for qPCR analysis cannot be designed due to their short cDNA sequences or high homologies with other genes in tomato. Thus, a total of 38 candidate RGs, which are designated as *SlFRG01* to *SlFRG38*, were eventually chosen for further expression validation in the qPCR experiments (Table 2).

qPCR amplification of the 38 candidate RGs were carried out using specific primers listed in Table 2, and the amplicon lengths ranged from 67 bp (*SlFRG21*) to 149 bp (*SlFRG07*). PCR-amplification specificity of each primer pair was verified by 1.5% agarose gel electrophoresis using cDNA templates (Supplementary Figure 1), and the melting curve analysis also showed single product peak (Supplementary Figure 2), which both confirm the specificities of the primer pairs. The amplification efficiencies (E) of these candidate RGs were found to vary from 0.76 (*SlFRG01*) to 1.39 (*SlFRG22*), and E values of more than 50% primer pairs (20/38) were ranged from 0.9 to 1.1, indicating their qualifications as primer pairs (Tichopad et al., 2002; Chung et al., 2004). Notably, the amplification efficiencies (E) of *SlFRG07* and *SlFRG13* could not be calculated due to their low transcript level in tomato fruit (Supplementary Table 4). The correlation coefficients (*R*^2^) of the 38 candidate RGs ranged from 0.915 (*SlFRG37*/*SlFRG38*) to 1 (*SlFRG06*/*SlFRG33*) (Table 2).

**Figure 2:**
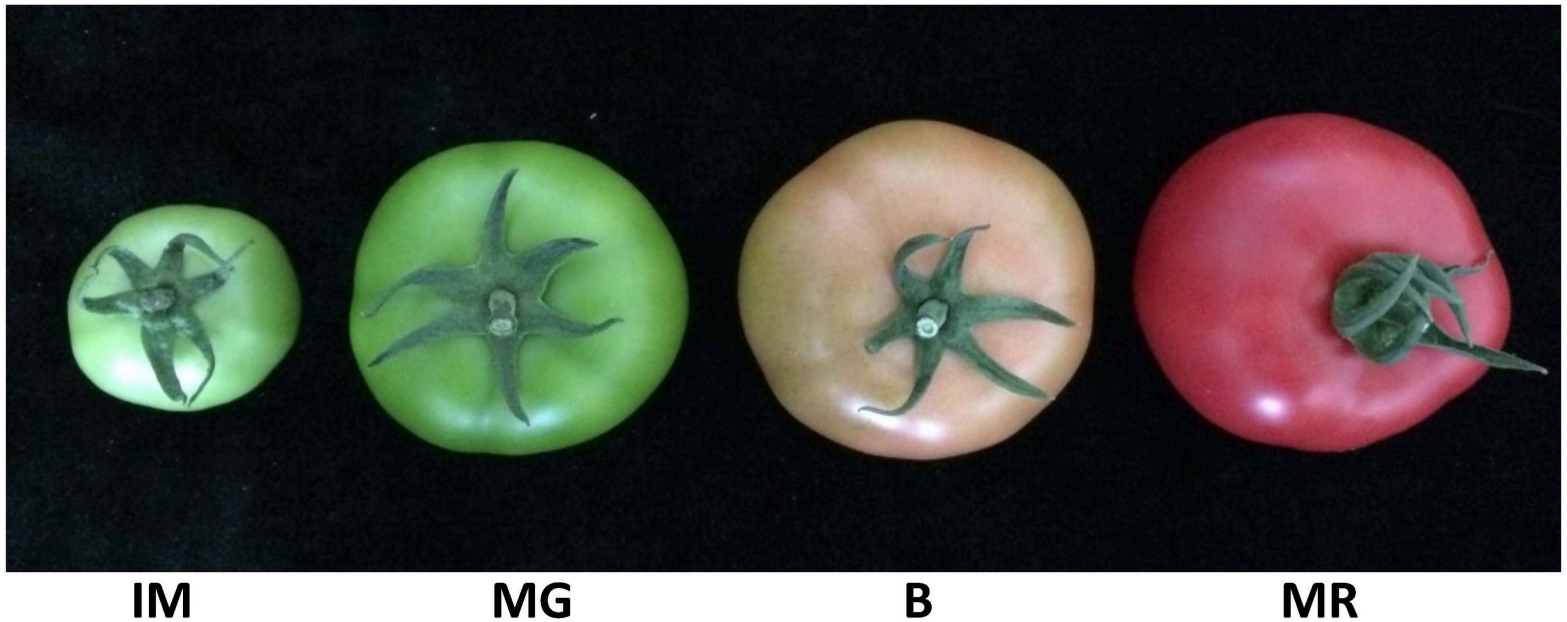
Tomato fruit samples of four representative developmental stages. IM, Immature, 15 days after fertilization, 5 cm diameter fruit; MG, Mature green, 30 days after fertilization, 9 cm diameter fruit; B, Breaker, 35 days after fertilization, 10 cm diameter fruit; MR, Mature red, 45 days after fertilization, 10 cm diameter fruit.

The Ct values of the each putative RG derived from different tomato fruit developmental stages (Immature-IM; Mature Green-MG; Breaker-B; Mature Red-MR) (Figure 2; Supplementary Table 4) were used here to evaluate the expression levels. The average Ct values of most candidate RGs (36/38) in various fruit developmental samples ranged between 20 and 30 (Except for *SlFRG07* [33.91] and *SlFRG13* [32.29]) (Supplementary Table 4).

### Validation of putative RGs using geNorm and normfinder

**Figure 3:**
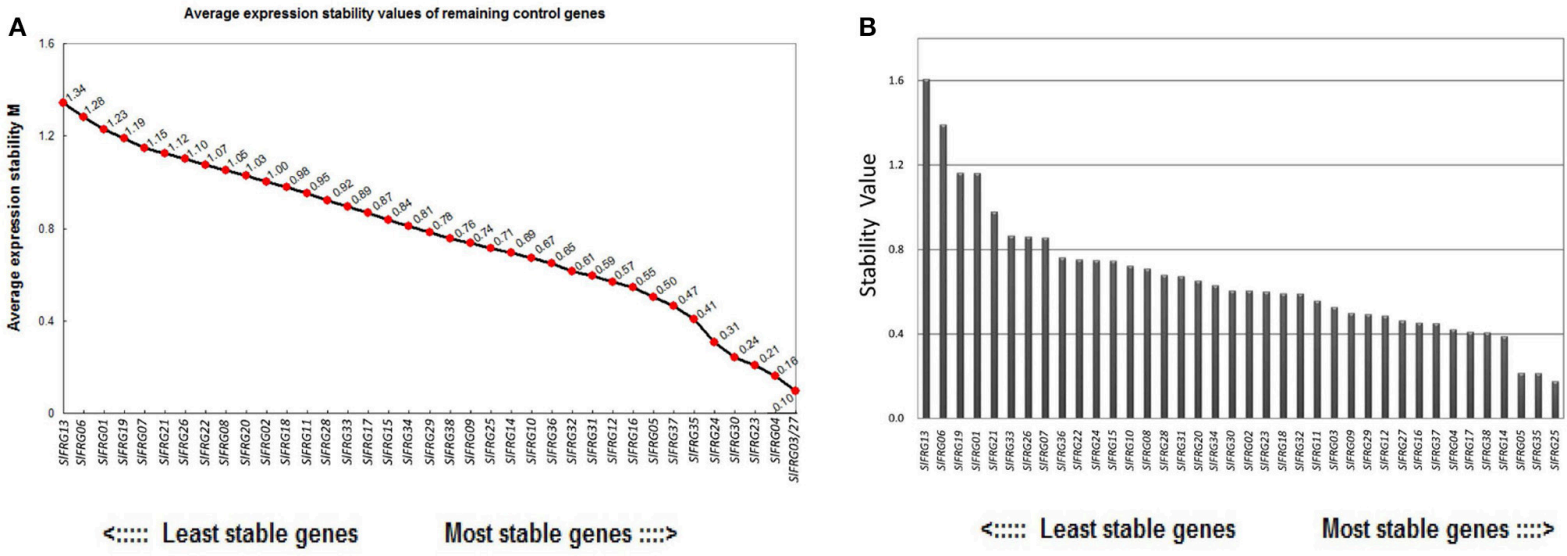
Expression stability of the 38 newly identified RGs evaluated by geNorm **(A)** and NormFinder **(B). (A)** Ranking of geNorm is based on the principle that logarithmically transformed gene expression ratio between two ideal internal control genes should be identical if both genes are stably expressed in the tested sample set. Expression stability values (M) of the 38 candidate RGs are shown, RGs with a higher M value are less stably expressed. **(B)** NormFinder is a model-based approach that evaluates expression variation by comparing the variation within and between a certain number of sample groups and RGs with lower combined levels of intra and intra-group variation were regarded to be more stably expressed.

Of all the 38 tested RGs, the geNorm analysis determined that the 12 most stable candidate RGs with average expression stabilities (M-value) less than 0.6, which were:*SlFRG03, SlFRG27, SlFRG04, SlFRG23, SlFRG30, SlFRG24, SlFRG35, SlFRG37, SlFRG05, SlFRG16, SlFRG12*, and *SlFRG31* (Figure 3A; Table 3). To ensure the evaluation results, Normfinder was also conducted for evaluation, and 12 RGs with expression stabilities (M-value) less than 0.5 were identified, which were: *SlFRG25, SlFRG35, SlFRG05, SlFRG14, SlFRG38, SlFRG17, SlFRG04, SlFRG37, SlFRG16, SlFRG27, SlFRG12*, and *SlFRG29* (Figure 3B; Table 3).

**Table 3:** Top-ranked RGs evaluated by geNorm and NormFinder.

**geNorm**	**M-value**	**NormFinder**	**M-value**	**Consensus**
*SlFRG03*	0.095	*SlFRG25*	0.177	*SlFRG27*
***SlFRG27***	0.095	***SlFRG35***	0.215	*SlFRG04*
***SlFRG04***	0.156	***SlFRG05***	0.216	*SlFRG35*
*SlFRG23*	0.206	*SlFRG14*	0.390	*SlFRG05*
*SlFRG30*	0.242	*SlFRG38*	0.408	*SlFRG37*
*SlFRG24*	0.305	*SlFRG17*	0.411	*SlFRG16*
***SlFRG35***	0.409	***SlFRG04***	0.423	*SlFRG12*
***SlFRG37***	0.467	***SlFRG37***	0.452	
***SlFRG05***	0.504	***SlFRG16***	0.454	
***SlFRG16***	0.547	***SlFRG27***	0.464	
***SlFRG12***	0.569	***SlFRG12***	0.487	
*SlFRG31*	0.594	*SlFRG29*	0.494	

*The seven RGs identified commonly in geNorm and Normfinder were labeled in bold*.

Although the two different assessing systems (geNorm and Normfinder) came up with different results, there were still seven putative RGs (*SlFRG27, SlFRG04, SlFRG35, SlFRG05, SlFRG37, SlFRG16, SlFRG12*) that were found to be commonly top-ranked in both statistical algorithms (Table 3). Generally speaking, the primer pairs with amplification efficiency (E) between 0.9 and 1.1 (Tiangen, China) possess the lowest variability in qPCR analysis (Tichopad et al., 2002; Chung et al., 2004). However, we found that among the seven top-ranked RGs, the amplification efficiencies (E) of two primer pairs (*SlFRG04* [0.78] and *SlFRG35* [0.87]) were lower than 0.9, suggesting that the primer pairs of these two genes were not recommended for subsequent RG application in this study. Ct values of the remaining five RGs were all between 20 and 25 (qualified as RG). Therefore, *SlFRG27, SlFRG05, SlFRG37, SlFRG16*, and *SlFRG12* were finally identified as qualified and optimal RGs for normalization in the tomato fruit developmental process.

**Figure 4:**
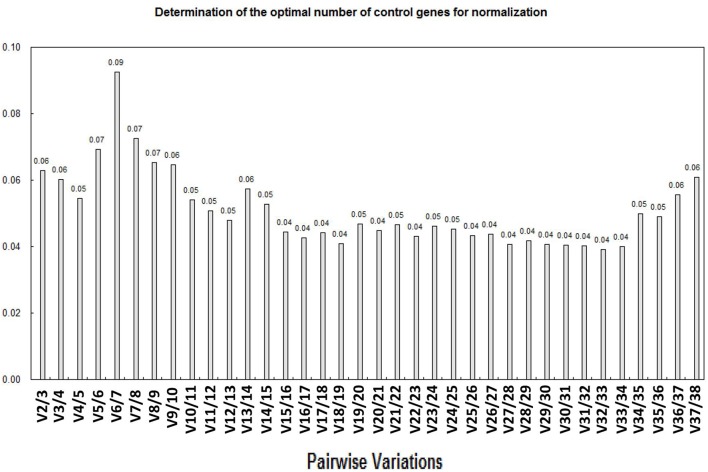
Analysis of best RG association based on geNorm algorithm. The optimum number of RGs is the lowest number of genes with an acceptably low Vn/n+1. Vandesompele et al. (2002) suggested 0.15 (15% variation in normalization factors) to be an upper limit for Vn/n+1. According to variations (V-value) calculation, V2/3 is 0.06 (<0.15), which means the most stable expressed RGs identified ingeNorm, *SlFRG03* and *SlFRG27*, are well-qualified as RG combination for normalization.

Previously, some researchers have reported that the use of more than one internal control genes in normalization could effectively improve the reliability of qPCR results (Reid et al., 2006; Exposito-Rodriguez et al., 2008; Gutierrez et al., 2008). Thus, we applied the geNorm software to calculate the pairwise variation values (V) of the 38 putative RGs (described in detailed in the Materials and Methods section). The pairwise variation revealed that the V2/3 value was 0.06 (significantly < 1.5) (Figure 4), which indicated that the combined use of two most stably expressed RGs as reflected in geNorm, *SlFRG03* and *SlFRG27*, was potentially sufficient for better normalization in qPCR experiments of tomato fruit developmental studies (Figure 4).

## Discussion

The advent of qPCR technology has brought a new revolution in the gene expression analysis area. Accurate interpretation of qRCR results mainly depends on the use of stable RGs for normalization, which can potentially minimize non-biological variations of different samples. Hence, the systematic identification of appropriate RGs is essential for obtaining reliable results in qPCR experiments (Udvardi et al., 2008; Bustin et al., 2009; Guenin et al., 2009). Nowadays, some HKGs (e.g., Actin, Ubiquitin, and 18s rRNA), are usually used as RGs under various experimental conditions, or across a broad range of tissue samples (Bustin, 2002; Kong et al., 2014). However, an increasing number of evidence showed that optimal RGs varied depending on the experimental conditions or organs/tissues assayed, and it seems to be impossible to acquire a list of RGs universally practicable across a wide range of experimental conditions (Guenin et al., 2009; Warzybok and Migocka, 2013; Kong et al., 2014). Therefore, the identification of suitable RGs for specific experimental conditions is essential for avoiding unnecessary error in the qRCR experimental results. So far, many studies involving the identification or evaluation of tomato RGs under various experimental conditions, including biotic/abiotic stresses (*Cucumber mosaic virus, tobacco mosaic virus, bacterium Xanthomonas campestris*, nitrogen stress, cold, light stress) (Coker and Davies, 2003; Løvdal and Lillo, 2009; Mascia et al., 2010; Wieczorek et al., 2013; Müller et al., 2015) and different organs/tissues (Leaf, fruit, flower, and seed) (Exposito-Rodriguez et al., 2008; Dekkers et al., 2012; Gao et al., 2012; Baldassarre et al., 2015), had been conducted. For example, *SlACT, SlCAC*, and *SlEF1*α were validated to be suitable RGs in studies of host-virus interactions in tomato (Wieczorek et al., 2013). Exposito-Rodriguez et al. (2008) found that the widely used RGs, such as *SlCAC, SlTIP41, Expressed*, and *SlSAND*, provide superior transcript normalization in various tissues of tomato (Exposito-Rodriguez et al., 2008). When studying the changes of gene expression in the wounded ripening-stage tomato fruit, Baldassarre et al. (2015) selected two most stably expressed RGs (*EF1-*α and *GADPH*) from seven routine used HKGs for normalization in the subsequent qPCR analysis. Thus, it occurred to us that although tomato is regarded as model plant for fruit development study, little attention has yet been paid to screen the best RGs specifically for normalization during the development of tomato fruit.

So far, most studies involving RG identification were based on the evaluation or validation of the expression stabilities of traditional or novel RGs under corresponding conditions (Czechowski et al., 2005; Løvdal and Lillo, 2009; Schmidt and Delaney, 2010; Dekkers et al., 2012; Baldassarre et al., 2015). In the present study, we collected 70 putative RGs that had been previously reported in tomato (Coker and Davies, 2003; Exposito-Rodriguez et al., 2008; Løvdal and Lillo, 2009; Mascia et al., 2010; Müller et al., 2015) and 11 orthologs of reported RGs in watermelon fruit study (Kong et al., 2015), and subsequently validated their expression stabilities during different stages of fruit development according to the RPKM values derived from RNA-seq data sets. Out of expectation, the majority of these putative RGs identified previously were not well-qualified for normalization as internal control genes due to either their unstable expressions or low transcript levels (Table 1; Figure 1A; Supplementary Table 1). Therefore, we next intended to identify some novel RGs that are stably expressed during the whole developmental process of tomato fruit.

Nowadays, the open sources of the SGN and TFGD allowed us to search for the most stably expressed genes on a comprehensive evaluation system. In the present study, we conducted a data mining based on the RNA-seq data set of different tomato fruit developmental stages, and initially collected 38 most stably expressed genes as putative RGs for normalization in tomato fruit developmental study (Table 2). Further evaluation of these 38 putative RGs was conducted using qPCR analysis in four developmental stages of tomato fruit (IM, MG, B, and MG) (Figure 2). Next, we used two popular statistical algorithms for RG ranking, geNorm, and Normfiner (Vandesompele et al., 2002; Andersen et al., 2004), for the RG evaluation based on the qPCR results (Supplementary Table 4). We found different ranking results from the evaluation results of geNorm and Normfinder (Table 3), which were explicable as these two algorithms are based on different models and assumptions (Schmidt and Delaney, 2010). The geNorm algorithm is based on the principle that logarithmically transformed expression ratio of two genes should be constant if both of them are stably expressed in the tested sample set. The relative stability of each gene (M) is defined as the mean pairwise variation (reflected by standard deviation of the expression ratios of two genes) of the gene in the sample set. Furthermore, as normalization with single RG can cause inevitable errors, geNorm is also used to determine the minimum number of RGs required for more reliable normalization (Vandesompele et al., 2002). Normfinder measures gene expression stability by comparing the variation within and between a certain number of sample groups. The genes with the lowest combined levels of intra and inter-group variation were regarded as most stably expressed (Andersen et al., 2004; Schmidt and Delaney, 2010). Taken together, Normfinder is based on analyzing the variation level of each tested gene rather than pairwise analysis of gene stability relative to a set of potential RGs (Schmidt and Delaney, 2010). So far, numerous ranking differences of RGs derived by these two algorithms had been found in many previous studies (Schmidt and Delaney, 2010; Cassan-Wang et al., 2012; Kong et al., 2015). Nevertheless, we identified 7 putative RGs (*SlFRG04, SlFRG35*,*SlFRG27, SlFRG05, SlFRG37, SlFRG16*, and *SlFRG12*) that were common in geNorm and Normfinder. Considering the unqualified primer amplification efficiencies (E) of *SlFRG04* and *SlFRG35*, these two genes were excluded from our recommendation list, and the remaining five genes (*SlFRG05, SlFRG12, SlFRG16, SlFRG27*, and *SlFRG37*) were finally identified as suitable internal controls for normalization in tomato fruit development. Notably, we believe that some alternative primer pairs of *SlFRG04* and *SlFRG35* with improved amplification efficiencies might be redesigned for RG use in tomato fruit developmental study.

In practice, it is believed that the use of more than one RG in the normalization can efficiently improve the reliability of qPCR results (Alba and Giovannoni, 2005; Exposito-Rodriguez et al., 2008; Gutierrez et al., 2008). Thus, in order to explore the minimun number of RGs needed, the pairwise variation (V) values were calculated in geNorm (Figure 4). According to the evaluation, the combined application of two RGs, *SlFRG03*, and *SlFRG27*, would be a better choice than the use of only one RG for normalization when more reliable qPCR results are needed. It is also worth noting that due to the multiple sections of tomato fruit and the complex biological processes of fruit development, gene expression analysis has been extended to more precise tissue parts (e.g., pericarp, flesh, and even seeds) or longer developmental stages of fruits (Fraser et al., 1994; Carrari and Fernie, 2006; Fei et al., 2011; Cheng et al., 2016). Therefore, we propose that the RGs identified in this study should be further validated in different tissue sections or earlier developmental stages (e.g., 1, 2 cm green fruits) of tomato fruit in the future according to specific experimental requirements.

## Conclusion

To our knowledge, this study is the first systematic identification and evaluation of putative RGs as internal controls for normalization of qPCR analysis in tomato fruit developmental process. According to our extensive evaluation, five identified RGs-*SlFRG05, SlFRG12, SlFRG16, SlFRG27*, and *SlFRG37* could be recommended for normalization of qPCR experiments in tomato fruits. Furthermore, according to geNorm analysis, a combination of two most stably expressed RGs, *SlFRG03* and *SlFRG27*, were recommended when more reliable qPCR results were needed. Moreover, by comparative analysis of the previously published materials involving RG identification for fruit developmental study in other plants, we found that two RGs identified in this study were also chosen as optimal RGs for fruit developmental study in other plants (Zhu et al., 2012; Die and Rowland, 2013; Kong et al., 2015), which are ubiquitin conjugating enzyme (UBI) encoding genes (*SlFRG27* in tomato, *PEX4* and *UBC28* in blueberry, *UBCE* in papaya) and actin encoding genes (*SlFRG37* in tomato, *ClACT* in watermelon, *ACTIN* in papaya). Thus, *SlFRG27*/*SlFRG37* and their corresponding orthologs seem to be universally applicable as RGs among plants of different families including Cucurbitaceae, Rosaceae, Vacciniaceae, and Solanacea. Taken together, the results presented here not only unveil optimal RGs for qPCR analysis in tomato fruit development, but also provide referable guidelines for identification of RGs in other plant species.

## Author contributions

Conceived and designed the experiments: HW and YC; Performed the experiments: YC, WB, XP, JY, MR, QY, RW, ZL, GZ, and ZY; Analyzed the data: JY, WB, XP and GZ; Wrote the paper: YC, WB, XP, GA, and HW. All authors have read and approved the manuscript.

### Conflict of interest statement

The authors declare that the research was conducted in the absence of any commercial or financial relationships that could be construed as a potential conflict of interest.

## Supplementary material

Supplementary Figure 1 PCR amplification products of the newly identified RGs on cDNA templates.

Supplementary Figure 2 Specificity of the newly identified RG primers demonstrated in qPCR analysis. Melting curves generated for all amplicons.

Supplementary Table 1 Expression levels (RPKM-value) of previoulsly reported RGs against RNA-seq data of different developmental stages of tomato fruit.

Supplementary Table 2 Expression levels (RPKM-value) of newly identified RGs against RNA-seq data of different developmental stages of tomato fruit.

Supplementary Table 3 CV (Co-efficient variation), average expression (AVE) and standard deviation (SD) values of previous reported RGs and newly identified RGs in tomato.

Supplementary Table 4 Ct-values of 38 newly identified RGs during tomato fruit development (IM, MG, B, MR).

Supplementary Table 5 Expression stability (M) values of 38 newly identified RGs evaluated by geNorm and NormFinder.
