# Supplementary figures and images for: Genome-Wide Identification and Evaluation of Reference Genes for Quantitative RT-PCR Analysis during Tomato Fruit Development
Source: Front Plant Sci. 2017 Aug 29;8:1440. doi: 10.3389/fpls.2017.01440 (PMC5581943; doi:10.3389/fpls.2017.01440)

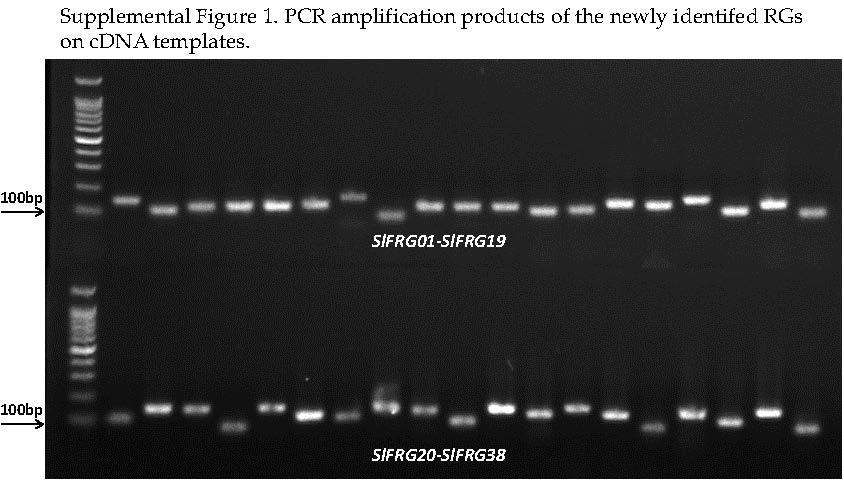

Supplement: Supplementary Figure 1 — PCR amplification products of the newly identified RGs on cDNA templates. [file Image1.JPEG]
