# Supplementary material for: Genome-Wide Identification and Evaluation of Reference Genes for Quantitative RT-PCR Analysis during Tomato Fruit Development
Source: Front Plant Sci. 2017 Aug 29;8:1440. doi: 10.3389/fpls.2017.01440 (PMC5581943; doi:10.3389/fpls.2017.01440)
Supplement: Supplementary Table 1 — Expression levels (RPKM-value) of previoulsly reported RGs against RNA-seq data of different developmental stages of tomato fruit. [file Table1.DOCX]

| Supplemental Table 1. Expression levels (RPKM value) of previoulsly reported RGs against RNA-seq data of different developmental stages of tomato fruit | | | | | | | | | |
| --- | --- | --- | --- | --- | --- | --- | --- | --- | --- |
| Gene ID | Heinz-1cm | Heinz-2cm | Heinz-3cm | Heinz-MG | Heinz-B | Heinz-B10 | Pimp-IM | Pimp-B | Pimp-B5 |
| Solyc05g014470 | 1687.48 | 1459.43 | 745.49 | 729.21 | 682.58 | 503.08 | 377.15 | 293.81 | 267.34 |
| [Solyc06g005060](https://solgenomics.net/tools/blast/show_match_seq.pl?blast_db_id=224;id=Solyc06g005060.2;hilite_coords=483-1701) | 1393.28 | 1165.85 | 1470.17 | 1476.39 | 1160.16 | 1231.98 | 733.95 | 642.03 | 517.99 |
| [Solyc10g006580](https://solgenomics.net/tools/blast/show_match_seq.pl?blast_db_id=224;id=Solyc10g006580.2;hilite_coords=111-926) | 523.71 | 263.7 | 350.96 | 928.73 | 776.16 | 1812.98 | 738.88 | 513.16 | 525.71 |
| Solyc11g006460 | 649.63 | 719.94 | 1588.97 | 1342 | 1505.29 | 797.03 | 964.61 | 799.02 | 732.38 |
| [Solyc09g074220](https://solgenomics.net/tools/blast/show_match_seq.pl?blast_db_id=224;id=Solyc09g074220.1;hilite_coords=137-249) | 0 | 0 | 0 | 0 | 0 | 0 | 0 | 0 | 0 |
| [Solyc11g005330](https://solgenomics.net/tools/blast/show_match_seq.pl?blast_db_id=222;id=Solyc11g005330.1;hilite_coords=21-356) | 555.72 | 535.28 | 719.64 | 819.55 | 802.91 | 608.38 | 472.74 | 361.2 | 354.52 |
| [Solyc12g096750](https://solgenomics.net/tools/blast/show_match_seq.pl?blast_db_id=224;id=Solyc12g096750.1;hilite_coords=1-1164) | 0 | 0 | 0 | 0 | 0 | 0 | 0 | 1.19 | 0 |
| [Solyc10g055810](https://solgenomics.net/tools/blast/show_match_seq.pl?blast_db_id=224;id=Solyc10g055810.1;hilite_coords=1-969) | 84.27 | 71.23 | 56.46 | 407.14 | 873.48 | 57.27 | 66.57 | 155.6 | 168.23 |
| Solyc07g066610 | 276.95 | 283.42 | 174.67 | 125.3 | 84.21 | 75.81 | 88.71 | 57.72 | 77.8 |
| [Solyc01g028810](https://solgenomics.net/feature/17675347/details) | 650.24 | 543.77 | 511.04 | 266.7 | 349.42 | 657.66 | 345.17 | 505.96 | 755.24 |
| [Solyc04g009770](https://solgenomics.net/feature/17787362/details) | 316.99 | 276.5 | 451.45 | 325.6 | 401.59 | 274.41 | 387.31 | 343.86 | 440.97 |
| [Solyc06g072120](https://solgenomics.net/feature/17857263/details) | 243.2 | 147.38 | 117.33 | 140.61 | 109.15 | 136.96 | 68.98 | 77.31 | 68.17 |
| Solyc06g061150 | 31.7 | 29.63 | 16.73 | 30 | 25.93 | 26.3 | 21.83 | 24.98 | 23.78 |
| [Solyc01g011340](https://solgenomics.net/tools/blast/show_match_seq.pl?blast_db_id=222;id=Solyc01g011340.2;hilite_coords=1-306) | 42.2 | 34.95 | 20.63 | 38.51 | 34.71 | 32.51 | 37.44 | 36.05 | 27.78 |
| [Solyc05g023800](https://solgenomics.net/tools/blast/show_match_seq.pl?blast_db_id=222;id=Solyc05g023800.2;hilite_coords=1-221) | 306.27 | 385.46 | 304.05 | 435.96 | 478.4 | 355.77 | 276.78 | 321.76 | 374.43 |
| [Solyc06g053820](https://solgenomics.net/tools/blast/show_match_seq.pl?blast_db_id=222;id=Solyc06g053820.2;hilite_coords=1-151) | 119.92 | 67.65 | 85.31 | 39.63 | 31.95 | 34.47 | 15.56 | 14.54 | 11.59 |
| [Solyc03g115810](https://solgenomics.net/tools/blast/show_match_seq.pl?blast_db_id=222;id=Solyc03g115810.2;hilite_coords=100-602) | 17.63 | 21.56 | 30.06 | 22.34 | 16.3 | 19.27 | 22.47 | 22.83 | 24.53 |
| [Solyc01g028930](https://solgenomics.net/tools/blast/show_match_seq.pl?blast_db_id=222;id=Solyc01g028930.2;hilite_coords=55-200) | 25.14 | 24.71 | 19.51 | 20.37 | 15.25 | 19.46 | 15.56 | 17.66 | 19.87 |
| [Solyc10g049850](https://solgenomics.net/tools/blast/show_match_seq.pl?blast_db_id=222;id=Solyc10g049850.1;hilite_coords=1-286) | 76.47 | 54.26 | 48.38 | 58.2 | 48.01 | 50.36 | 60.75 | 60.08 | 57.08 |
| [Solyc02g087880](https://solgenomics.net/tools/blast/show_match_seq.pl?blast_db_id=222;id=Solyc02g087880.2;hilite_coords=1-450) | 63.23 | 7.52 | 3.45 | 1.58 | 0.51 | 0.27 | 1.93 | 1.34 | 0.14 |
| [Solyc04g081490](https://solgenomics.net/tools/blast/show_match_seq.pl?blast_db_id=222;id=Solyc04g081490.2;hilite_coords=1-433) | 675.4 | 565.94 | 535.92 | 519.48 | 159.96 | 18.95 | 210.79 | 95.51 | 14.81 |
| [Solyc09g008700](https://solgenomics.net/tools/blast/show_match_seq.pl?blast_db_id=222;id=Solyc09g008700.1;hilite_coords=650-1032) | 3.93 | 7.14 | 11.55 | 11.06 | 9.9 | 12.03 | 16.54 | 13.52 | 16.73 |
| Solyc05g007050 | 16.29 | 15.21 | 22.7 | 13.01 | 7.97 | 5.85 | 23.11 | 20.49 | 11.48 |
| Solyc03g078400 | 780.22 | 887.94 | 1555.14 | 1488.54 | 935.42 | 897.86 | 831.89 | 581.51 | 467.27 |
| Solyc01g108340 | 129.24 | 150.85 | 83.29 | 50.26 | 9.99 | 12.19 | 32.69 | 19.25 | 15.23 |
| Solyc03g111010 | 102.66 | 111.91 | 112.79 | 164.02 | 248.11 | 322.68 | 355.97 | 423.69 | 254.7 |
| Solyc01g056940 | 845.61 | 444.48 | 448.2 | 380.43 | 319.91 | 280.89 | 182.49 | 161.27 | 114.92 |
| Solyc01g088480 | 84.68 | 56.14 | 26.65 | 53.36 | 12.83 | 7.43 | 27.05 | 13.52 | 4.19 |
| Solyc11g051210 | 0 | 0 | 0 | 0 | 0 | 0 | 0 | 0 | 0 |
| Solyc08g006960 | 27.11 | 27.45 | 17.41 | 24.28 | 21.75 | 25.65 | 30.36 | 31.12 | 20.77 |
| Solyc07g025390 | 12.89 | 10.38 | 10.3 | 13.4 | 8.05 | 14.91 | 14.31 | 13.65 | 11.01 |
| Solyc10g006100 | 11.85 | 13.62 | 11.22 | 12.33 | 10.38 | 8.07 | 18.05 | 15.55 | 12.75 |
| Solyc07g062920 | 11.83 | 11.86 | 10.28 | 11.42 | 8.17 | 10.47 | 18.9 | 22.9 | 18.88 |
| Solyc01g111780 | 48.03 | 45.41 | 65.42 | 63.28 | 56.88 | 48.69 | 44.45 | 46.75 | 46.51 |
| Solyc06g051420 | 38.18 | 32.08 | 37.41 | 28.85 | 28.5 | 32.99 | 32.03 | 33.36 | 31.01 |
| Solyc12g057120 | 40.93 | 35.55 | 41.25 | 51.14 | 76.49 | 97.03 | 41.17 | 44.57 | 52.37 |
| Solyc01g009290 | 11.95 | 9.62 | 11.19 | 10.84 | 11.33 | 14.95 | 14.98 | 13.4 | 15.51 |
| Solyc09g018730 | 54.76 | 45.36 | 53.7 | 34.05 | 31.03 | 30.49 | 39.06 | 36.23 | 39.55 |
| Solyc02g088110 | 23.77 | 27.25 | 30.86 | 26.42 | 22.3 | 32.31 | 15.84 | 17.56 | 21.25 |
| Solyc08g060860 | 22.86 | 23.55 | 22.47 | 13.94 | 16.96 | 14.09 | 11.38 | 11.47 | 9.29 |
| Solyc09g009640 | 138.43 | 112.33 | 130.81 | 83.94 | 57.35 | 54.66 | 78.92 | 63.24 | 53.18 |
| Solyc04g015370 | 150.37 | 112.85 | 149.67 | 115.42 | 96.8 | 99.19 | 81.91 | 99 | 114.81 |
| Solyc08g005140 | 7.97 | 6.79 | 10.35 | 6 | 5.31 | 10.18 | 9.14 | 10.22 | 12.36 |
| Solyc02g062920 | 42.25 | 31.94 | 52.7 | 43.33 | 39.41 | 40.12 | 45.23 | 33.98 | 32.09 |
| Solyc10g076910 | 10.74 | 14.64 | 15.97 | 16.68 | 25.01 | 24.29 | 29.78 | 28.5 | 30.03 |
| Solyc03g121980 | 42.52 | 41.25 | 48.25 | 63.39 | 44.54 | 42.99 | 51.57 | 45.6 | 37.55 |
| Solyc01g097140 | 34.93 | 25.72 | 22.81 | 32.82 | 28.25 | 20.34 | 36.43 | 36.84 | 29.51 |
| Solyc07g007040 | 30.93 | 34.23 | 42.42 | 34.46 | 29.16 | 39.54 | 50.21 | 38.62 | 17.88 |
| Solyc06g069310 | 50.4 | 39.51 | 46.14 | 51.64 | 43.31 | 44.79 | 41.08 | 34.19 | 31.26 |
| Solyc03g078020 | 8.68 | 7.81 | 10.75 | 7.57 | 8.19 | 11.34 | 11.1 | 13.77 | 13.51 |
| Solyc10g078180 | 47.81 | 51.29 | 74.19 | 56.12 | 60.83 | 58.45 | 50.58 | 59.8 | 63.93 |
| Solyc02g089230 | 36.11 | 31.62 | 20.18 | 33.59 | 23.2 | 16.91 | 28.62 | 32.3 | 24.9 |
| Solyc06g036720 | 19.52 | 19.22 | 35.02 | 33.57 | 32.96 | 32.18 | 35.63 | 29.28 | 36.61 |
| Solyc01g109620 | 123.31 | 99.96 | 97.14 | 87.49 | 79.43 | 109.44 | 91.53 | 96.17 | 81.49 |
| Solyc07g064510 | 116.92 | 108.98 | 168.66 | 176.6 | 186.72 | 121.03 | 115.23 | 118.92 | 124.12 |
| Solyc11g071930 | 25.21 | 22.35 | 21.87 | 25.7 | 35.13 | 39.71 | 23.41 | 29.04 | 33.5 |
| Solyc06g084000 | 48.29 | 53.3 | 80 | 62.26 | 62.26 | 54.84 | 36.91 | 36.8 | 42.06 |
| Solyc04g009230 | 18.93 | 18.7 | 19.36 | 32.87 | 58.54 | 117.06 | 57.47 | 84.68 | 206.31 |
| Solyc06g073870 | 125.18 | 99.42 | 84.22 | 93.75 | 72.36 | 85.33 | 73.4 | 79.56 | 66.31 |
| Solyc09g055760 | 39.1 | 31.24 | 28.88 | 32.82 | 20.96 | 23.33 | 27.49 | 24.25 | 23.45 |
| Solyc12g005780 | 27.88 | 25.8 | 37.78 | 34.19 | 26.98 | 34.19 | 26.76 | 23.06 | 18.99 |
| Solyc04g008610 | 4.66 | 8.41 | 11.1 | 11.23 | 11.46 | 12.04 | 13.57 | 13.02 | 11.53 |
| Solyc04g015300 | 10.41 | 9.55 | 7.24 | 7.1 | 4.23 | 5.21 | 9.5 | 6.94 | 6.09 |
| Solyc10g005800 | 104.33 | 111.51 | 166.84 | 119.99 | 127.64 | 95.33 | 106.1 | 108.24 | 119.7 |
| Solyc12g021130 | 16 | 19.77 | 31.72 | 22.51 | 17.15 | 14.48 | 23.75 | 21.18 | 19.09 |
| Solyc01g079330 | 12.17 | 13.76 | 15.38 | 16.45 | 13.5 | 18.59 | 25.46 | 21.89 | 19.71 |
| Solyc07g041550 | 25.21 | 20.65 | 25.82 | 20.8 | 15.62 | 20.19 | 22.06 | 17.06 | 16.62 |
| Solyc03g059420 | 6.82 | 7.85 | 9.87 | 10.6 | 10.24 | 11.55 | 12.05 | 11.53 | 13.76 |
| Solyc11g071950 | 22.95 | 19.31 | 27.78 | 17.7 | 11.39 | 14.86 | 21.15 | 15.64 | 17.65 |
| Solyc12g099570 | 43.63 | 26.64 | 34.16 | 29.73 | 35.61 | 22.16 | 13.48 | 2.82 | 18.36 |
| Solyc10g044900 | 20.04 | 12.27 | 17.74 | 13.59 | 7.22 | 9.55 | 7.26 | 7.38 | 3.04 |
| Solyc10g084270 | 13.6 | 12.27 | 12.16 | 11.51 | 9.8 | 13.23 | 19.44 | 16.44 | 14.02 |
| Solyc06g016750 | 26.5 | 33.95 | 33.77 | 24.21 | 24.66 | 25.53 | 20.99 | 19.79 | 19.67 |
| Solyc02g092380 | 12.61 | 10.92 | 10.1 | 11.89 | 8.66 | 13.01 | 12.12 | 10.66 | 6.69 |
| Solyc05g052960 | 20.3 | 18.21 | 18.2 | 16.13 | 13.43 | 22.83 | 20.01 | 20.76 | 19.89 |
| Solyc06g009860 | 0 | 0 | 0.21 | 0 | 0 | 0 | 0 | 0 | 0 |
| Solyc10g008950 | 9.8 | 13.56 | 18.04 | 11.94 | 9.16 | 12.04 | 16.07 | 12.32 | 13.24 |
| Solyc10g055450 | 11.81 | 18.79 | 22.49 | 27.91 | 25.94 | 31.16 | 30.24 | 32.68 | 31.79 |
| Solyc05g006580 | 9.33 | 7.73 | 8.78 | 10.52 | 9.43 | 11.19 | 15.29 | 14.23 | 11.94 |
| Solyc03g121310 | 79.77 | 67.83 | 88.35 | 101.24 | 80.39 | 60.89 | 104.87 | 115.77 | 86.3 |
| Solyc09g010180 | 23.91 | 30.66 | 42.9 | 44.96 | 33.55 | 36.54 | 53.06 | 48.6 | 44.79 |

Heinz-1cm: 1cm diameter fruit of 'Heinz'; Heinz-2cm: 2cm diameter fruit of 'Heinz'; Heinz-3cm: 3cm diameter fruit of 'Heinz'; Heinz-MG: Mature green fruit of 'Heinz'; Heinz-B: Breaker fruit of 'Heinz'; Heinz-MG: Mature green fruit of 'Heinz'; Heinz-B10: 10days after breaker fruit of 'Heinz'; Pimp-IM: Immature fruit of 'Pimp'; Pimp-B: Breaker fruit of 'Pimp'; Pimp-B5: 5 days after Breaker fruit of 'Pimp'
