# Supplementary material for: Genome-Wide Identification and Evaluation of Reference Genes for Quantitative RT-PCR Analysis during Tomato Fruit Development
Source: Front Plant Sci. 2017 Aug 29;8:1440. doi: 10.3389/fpls.2017.01440 (PMC5581943; doi:10.3389/fpls.2017.01440)
Supplement: Supplementary Table 2 — Expression levels (RPKM-value) of newly identified RGs against RNA-seq data of different developmental stages of tomato fruit. [file Table2.DOCX]

| Supplemental Table 2. Expression levels (RPKM value) of newly identified RGs against RNA-seq data of different developmental stages of tomato fruit | | | | | | | | | |
| --- | --- | --- | --- | --- | --- | --- | --- | --- | --- |
| Gene ID | Heinz-1cm | Heinz-2cm | Heinz-3cm | Heinz-MG | Heinz-B | Heinz-B10 | Pimp-IM | Pimp-B | Pimp-B5 |
| Solyc06g005360 | 393.53 | 381.52 | 380.68 | 412.03 | 463.85 | 393.38 | 476.23 | 447.27 | 490.02 |
| Solyc01g088040 | 675.44 | 639.5 | 515.65 | 737.04 | 673.37 | 634.96 | 637.73 | 621.67 | 543.94 |
| Solyc02g063070 | 337.87 | 291.59 | 331.45 | 280.29 | 273.99 | 335.95 | 251.27 | 269.95 | 292.53 |
| Solyc12g095990 | 271.92 | 262.74 | 319.28 | 330.93 | 359.31 | 332.83 | 361.1 | 349.44 | 279.84 |
| Solyc01g104170 | 388 | 280.04 | 363.17 | 317.12 | 315.64 | 306.18 | 320.93 | 282.81 | 268.04 |
| Solyc11g070030 | 324.96 | 223.1 | 326.29 | 291.2 | 303.98 | 339.28 | 254.43 | 274.96 | 320.58 |
| Solyc01g091150 | 382.65 | 374.27 | 310.83 | 367.46 | 295.31 | 419.06 | 402.02 | 424.94 | 451.86 |
| Solyc11g042930 | 1061.36 | 1074.44 | 1214.02 | 1470.96 | 1214.05 | 984.65 | 1239.02 | 973.77 | 915.01 |
| Solyc03g031950 | 280.78 | 330.68 | 409.84 | 468.39 | 450.93 | 479.43 | 441.58 | 446.39 | 499.27 |
| Solyc10g078450 | 434.56 | 374.41 | 305.58 | 329.84 | 345.48 | 334.62 | 280.94 | 253.4 | 270.42 |
| Solyc05g023800 | 306.27 | 385.46 | 304.05 | 435.96 | 478.4 | 355.77 | 276.78 | 321.76 | 374.43 |
| Solyc04g009770 | 316.99 | 276.5 | 451.45 | 325.6 | 401.59 | 274.41 | 387.31 | 343.86 | 440.97 |
| Solyc01g103450 | 206.92 | 316.32 | 308.58 | 259.49 | 302.09 | 412.26 | 246.84 | 288.62 | 272.02 |
| Solyc06g076970 | 365.95 | 274.7 | 297.57 | 340.35 | 364.4 | 331.1 | 246.11 | 234.51 | 202.51 |
| Solyc01g010750 | 223.21 | 311.64 | 424.52 | 319.37 | 370.93 | 344.38 | 231.14 | 270.12 | 268.91 |
| Solyc10g081190 | 221.04 | 289.08 | 300.73 | 394.18 | 362.85 | 308.07 | 467.33 | 421.11 | 324.41 |
| Solyc04g056350 | 256 | 278.17 | 274.57 | 354.7 | 431.32 | 408.42 | 485.62 | 416.25 | 347.21 |
| Solyc01g104700 | 412.4 | 422.74 | 303.19 | 337.78 | 342.38 | 243.39 | 220.48 | 267.42 | 411.09 |
| Solyc10g006480 | 210.55 | 265.32 | 378.76 | 280.13 | 297.75 | 307.39 | 372.93 | 399.42 | 206.92 |
| Solyc10g083570 | 395.76 | 385.3 | 278.76 | 289.32 | 431.48 | 326.8 | 258.89 | 215.98 | 254.63 |
| Solyc02g089200 | 435.16 | 401.71 | 274.11 | 263.83 | 255.13 | 212.54 | 268.4 | 345.74 | 317.2 |
| Solyc08g081190 | 827.1 | 1080.58 | 1086.12 | 573.87 | 792.71 | 1339.38 | 878.1 | 813.82 | 905.14 |
| Solyc06g036540 | 202.97 | 217.05 | 287.98 | 389.07 | 402.58 | 366.97 | 338.04 | 362.7 | 252.66 |
| Solyc02g080630 | 295.14 | 228.74 | 247.8 | 215.73 | 267.08 | 296.57 | 394.5 | 433.17 | 317.56 |
| Solyc05g050200 | 445.02 | 262.07 | 324.22 | 216.9 | 280.27 | 272.71 | 204.49 | 234.33 | 223.61 |
| Solyc01g087320 | 208.35 | 201.5 | 298.36 | 286.55 | 261.99 | 333.15 | 443.38 | 407.02 | 473.96 |
| Solyc06g007510 | 702.22 | 710.67 | 1547.29 | 1306.17 | 1392.23 | 679.88 | 1555.79 | 1280.68 | 1345.05 |
| Solyc06g005060 | 1393.28 | 1165.85 | 1470.17 | 1476.39 | 1160.16 | 1231.98 | 733.95 | 642.03 | 517.99 |
| Solyc02g084360 | 200.72 | 326.16 | 502.82 | 429.85 | 368.48 | 448.15 | 299.3 | 290.31 | 218.66 |
| Solyc09g082650 | 263.41 | 234.5 | 284.22 | 285.06 | 370.48 | 395.07 | 501.31 | 618.8 | 466.18 |
| Solyc12g044600 | 314.68 | 486.11 | 409.34 | 529.02 | 483.29 | 597.06 | 258.15 | 237.64 | 384.34 |
| Solyc01g107870 | 319.66 | 370.47 | 428.45 | 435.49 | 506.69 | 365.08 | 400.78 | 344.35 | 269.54 |
| Solyc12g055800 | 361.65 | 546.94 | 530.65 | 448.99 | 337.5 | 333.44 | 458.22 | 343.76 | 203.99 |
| Solyc12g008590 | 405.71 | 340.23 | 270.8 | 395.85 | 393.66 | 402.11 | 430 | 503.21 | 597.23 |
| Solyc01g095050 | 425.2 | 401.97 | 493.46 | 715.16 | 569.02 | 267.27 | 499.91 | 431.09 | 255.16 |
| Solyc02g085540 | 439.59 | 310.44 | 562.49 | 384.56 | 521.68 | 406.45 | 257.08 | 229.01 | 323.35 |
| Solyc09g009260 | 517.83 | 513.72 | 262.95 | 531.39 | 824.61 | 868.08 | 458.43 | 492.35 | 775.48 |
| Solyc11g005330 | 555.72 | 535.28 | 719.64 | 819.55 | 802.91 | 608.38 | 472.74 | 361.2 | 354.52 |
| Solyc01g028810 | 650.24 | 543.77 | 511.04 | 266.7 | 349.42 | 657.66 | 345.17 | 505.96 | 755.24 |
| Solyc10g074860 | 1062.78 | 473.02 | 581.07 | 391.66 | 433.19 | 604.36 | 742.62 | 637.81 | 545.38 |
| Solyc06g009970 | 1023.48 | 1077.19 | 1236.4 | 1298.18 | 939.11 | 890.17 | 585.94 | 530.14 | 425.14 |
| Solyc11g006460 | 649.63 | 719.94 | 1588.97 | 1342 | 1505.29 | 797.03 | 964.61 | 799.02 | 732.38 |
| Solyc07g066600 | 926.34 | 881.63 | 896.8 | 630.91 | 575.13 | 422.94 | 356.18 | 350.01 | 511.83 |
| Solyc01g067740 | 766.21 | 677.71 | 710.02 | 828.96 | 1477.22 | 1744.07 | 880.14 | 1113.91 | 1677.05 |
| Solyc03g078400 | 780.22 | 887.94 | 1555.14 | 1488.54 | 935.42 | 897.86 | 831.89 | 581.51 | 467.27 |
| Solyc07g065840 | 892.43 | 1111.2 | 1908.39 | 1486.98 | 1051.16 | 656.58 | 1032.38 | 853.14 | 557.57 |
| Solyc08g075690 | 1028.45 | 940.34 | 992.85 | 698.27 | 612.27 | 397.82 | 561.46 | 444.71 | 317.02 |
| Solyc07g066310 | 1579.64 | 791.83 | 975.71 | 656.91 | 734.07 | 491.06 | 858.84 | 980.16 | 1705.6 |
| Solyc11g070130 | 574.66 | 766.34 | 1278.67 | 1377.42 | 931.03 | 544.19 | 663.71 | 541.96 | 451.53 |
| Solyc01g099780 | 760.45 | 652.46 | 1361.13 | 1597.67 | 1435.8 | 671.73 | 828.52 | 711.85 | 460.67 |
| Solyc12g044720 | 978.91 | 929.7 | 1395.89 | 976.94 | 765.9 | 414.83 | 600.91 | 480.58 | 341.86 |
| Solyc06g009630 | 503.91 | 465.65 | 582.09 | 432.98 | 688.73 | 415.05 | 1173.39 | 976.83 | 1498.2 |
| Solyc06g071720 | 796.96 | 698.49 | 1468.64 | 1130.72 | 1251.95 | 559.86 | 428.61 | 357.24 | 336.33 |
| Solyc10g006580 | 523.71 | 263.7 | 350.96 | 928.73 | 776.16 | 1812.98 | 738.88 | 513.16 | 525.71 |
| Solyc05g014470 | 1687.48 | 1459.43 | 745.49 | 729.21 | 682.58 | 503.08 | 377.15 | 293.81 | 267.34 |
| Solyc10g076510 | 208.88 | 370.01 | 313.81 | 298.09 | 604.62 | 281.5 | 281 | 734.56 | 1282.26 |

Heinz-1cm: 1cm diameter fruit of 'Heinz'; Heinz-2cm: 2cm diameter fruit of 'Heinz'; Heinz-3cm: 3cm diameter fruit of 'Heinz'; Heinz-MG: Mature green fruit of 'Heinz'; Heinz-B: Breaker fruit of 'Heinz'; Heinz-MG: Mature green fruit of 'Heinz'; Heinz-B10: 10days after breaker fruit of 'Heinz'; Pimp-IM: Immature fruit of 'Pimp'; Pimp-B: Breaker fruit of 'Pimp'; Pimp-B5: 5 days after Breaker fruit of 'Pimp'
