# Supplementary material for: Genome-Wide Identification and Evaluation of Reference Genes for Quantitative RT-PCR Analysis during Tomato Fruit Development
Source: Front Plant Sci. 2017 Aug 29;8:1440. doi: 10.3389/fpls.2017.01440 (PMC5581943; doi:10.3389/fpls.2017.01440)
Supplement: Supplementary Table 3 — CV (Co-efficient variation), average expression (AVE) and standard deviation (SD) values of previous reported RGs and newly identified RGs in tomato. [file Table3.DOCX]

| Supplemental Tabel 3. CV (Co-efficient variation), average expression (AVE) and standard deviation (SD) values of previous reported RGs and newly identified RGs in tomato | | | | | | | |
| --- | --- | --- | --- | --- | --- | --- | --- |
| Published reference genes | CV | AVE | SD | Selected reference genes | CV | AVE | SD |
| Solyc05g014470 | 0.67 | 749.51 | 503.51 | Solyc06g005360 | 0.10 | 426.50 | 43.08 |
| Solyc06g005060 | 0.34 | 1087.98 | 365.93 | Solyc01g088040 | 0.11 | 631.03 | 67.19 |
| Solyc10g006580 | 0.65 | 714.89 | 461.64 | Solyc02g063070 | 0.11 | 296.10 | 31.72 |
| Solyc11g006460 | 0.36 | 1010.99 | 366.36 | Solyc12g095990 | 0.12 | 318.60 | 38.03 |
| Solyc09g074220 | 0.00 | 0.00 | 0.00 | Solyc01g104170 | 0.12 | 315.77 | 39.09 |
| Solyc11g005330 | 0.30 | 581.10 | 173.27 | Solyc11g070030 | 0.13 | 295.42 | 38.41 |
| Solyc12g096750 | 3.00 | 0.13 | 0.40 | Solyc01g091150 | 0.14 | 380.93 | 51.65 |
| Solyc10g055810 | 1.26 | 215.58 | 270.65 | Solyc11g042930 | 0.15 | 1127.48 | 173.84 |
| Solyc07g066610 | 0.63 | 138.29 | 87.48 | Solyc03g031950 | 0.17 | 423.03 | 72.16 |
| Solyc01g028810 | 0.32 | 509.47 | 163.90 | Solyc10g078450 | 0.17 | 325.47 | 56.35 |
| Solyc04g009770 | 0.18 | 357.63 | 66.08 | Solyc05g023800 | 0.18 | 359.88 | 66.21 |
| Solyc06g072120 | 0.44 | 123.23 | 54.52 | Solyc04g009770 | 0.18 | 357.63 | 66.08 |
| Solyc06g061150 | 0.18 | 25.65 | 4.61 | Solyc01g103450 | 0.20 | 290.35 | 57.17 |
| Solyc01g011340 | 0.19 | 33.86 | 6.37 | Solyc06g076970 | 0.20 | 295.24 | 59.41 |
| Solyc05g023800 | 0.18 | 359.88 | 66.21 | Solyc01g010750 | 0.22 | 307.14 | 66.20 |
| Solyc06g053820 | 0.79 | 46.74 | 36.99 | Solyc10g081190 | 0.22 | 343.20 | 75.59 |
| Solyc03g115810 | 0.19 | 21.89 | 4.05 | Solyc04g056350 | 0.22 | 361.36 | 80.13 |
| Solyc01g028930 | 0.18 | 19.73 | 3.47 | Solyc01g104700 | 0.23 | 328.99 | 76.08 |
| Solyc10g049850 | 0.15 | 57.07 | 8.74 | Solyc10g006480 | 0.23 | 302.13 | 70.43 |
| Solyc02g087880 | 2.31 | 8.89 | 20.51 | Solyc10g083570 | 0.23 | 315.21 | 73.94 |
| Solyc04g081490 | 0.84 | 310.75 | 260.80 | Solyc02g089200 | 0.24 | 308.20 | 73.34 |
| Solyc09g008700 | 0.36 | 11.38 | 4.12 | Solyc08g081190 | 0.24 | 921.87 | 219.98 |
| Solyc05g007050 | 0.41 | 15.12 | 6.19 | Solyc06g036540 | 0.24 | 313.34 | 75.33 |
| Solyc03g078400 | 0.39 | 936.20 | 366.21 | Solyc02g080630 | 0.24 | 299.59 | 73.31 |
| Solyc01g108340 | 0.95 | 55.89 | 53.30 | Solyc05g050200 | 0.27 | 273.74 | 74.29 |
| Solyc03g111010 | 0.51 | 232.95 | 117.84 | Solyc01g087320 | 0.30 | 323.81 | 98.74 |
| Solyc01g056940 | 0.62 | 353.13 | 220.70 | Solyc06g007510 | 0.31 | 1168.89 | 366.06 |
| Solyc01g088480 | 0.86 | 31.76 | 27.28 | Solyc06g005060 | 0.34 | 1087.98 | 365.93 |
| Solyc11g051210 | 0.00 | 0.00 | 0.00 | Solyc02g084360 | 0.30 | 342.72 | 103.36 |
| Solyc08g006960 | 0.18 | 25.10 | 4.52 | Solyc09g082650 | 0.34 | 379.89 | 128.66 |
| Solyc07g025390 | 0.19 | 12.10 | 2.27 | Solyc12g044600 | 0.30 | 411.07 | 123.91 |
| Solyc10g006100 | 0.23 | 12.65 | 2.90 | Solyc01g107870 | 0.18 | 382.28 | 70.08 |
| Solyc07g062920 | 0.36 | 13.86 | 5.04 | Solyc12g055800 | 0.28 | 396.13 | 109.38 |
| Solyc01g111780 | 0.16 | 51.71 | 8.03 | Solyc12g008590 | 0.22 | 415.42 | 92.65 |
| Solyc06g051420 | 0.10 | 32.71 | 3.33 | Solyc02g085540 | 0.30 | 486.08 | 344.41 |
| Solyc12g057120 | 0.38 | 53.39 | 20.29 | Solyc09g009260 | 0.34 | 582.76 | 198.30 |
| Solyc01g009290 | 0.17 | 12.64 | 2.13 | Solyc11g005330 | 0.30 | 581.10 | 173.27 |
| Solyc09g018730 | 0.22 | 40.47 | 9.04 | Solyc01g028810 | 0.32 | 509.47 | 163.90 |
| Solyc02g088110 | 0.23 | 24.17 | 5.60 | Solyc01g095050 | 0.32 | 450.92 | 142.95 |
| Solyc08g060860 | 0.34 | 16.22 | 5.49 | Solyc10g074860 | 0.33 | 607.99 | 201.89 |
| Solyc09g009640 | 0.39 | 85.87 | 33.36 |  |  |  |  |
| Solyc04g015370 | 0.21 | 113.34 | 23.36 |  |  |  |  |
| Solyc08g005140 | 0.27 | 8.70 | 2.34 |  |  |  |  |
| Solyc02g062920 | 0.17 | 40.12 | 6.79 |  |  |  |  |
| Solyc10g076910 | 0.34 | 21.74 | 7.30 |  |  |  |  |
| Solyc03g121980 | 0.16 | 46.41 | 7.54 |  |  |  |  |
| Solyc01g097140 | 0.20 | 29.74 | 5.98 |  |  |  |  |
| Solyc07g007040 | 0.26 | 35.27 | 9.11 |  |  |  |  |
| Solyc06g069310 | 0.16 | 42.48 | 6.81 |  |  |  |  |
| Solyc03g078020 | 0.23 | 10.30 | 2.37 |  |  |  |  |
| Solyc10g078180 | 0.14 | 58.11 | 8.03 |  |  |  |  |
| Solyc02g089230 | 0.24 | 27.49 | 6.55 |  |  |  |  |
| Solyc06g036720 | 0.22 | 30.44 | 6.63 |  |  |  |  |
| Solyc01g109620 | 0.14 | 96.22 | 13.79 |  |  |  |  |
| Solyc07g064510 | 0.22 | 137.46 | 30.52 |  |  |  |  |
| Solyc11g071930 | 0.22 | 28.44 | 6.34 |  |  |  |  |
| Solyc06g084000 | 0.26 | 52.97 | 14.00 |  |  |  |  |
| Solyc04g009230 | 0.90 | 68.21 | 61.70 |  |  |  |  |
| Solyc06g073870 | 0.21 | 86.61 | 17.85 |  |  |  |  |
| Solyc09g055760 | 0.21 | 27.95 | 5.75 |  |  |  |  |
| Solyc12g005780 | 0.21 | 28.40 | 5.96 |  |  |  |  |
| Solyc04g008610 | 0.25 | 10.78 | 2.71 |  |  |  |  |
| Solyc04g015300 | 0.28 | 7.36 | 2.09 |  |  |  |  |
| Solyc10g005800 | 0.18 | 117.74 | 20.81 |  |  |  |  |
| Solyc12g021130 | 0.25 | 20.63 | 5.14 |  |  |  |  |
| Solyc01g079330 | 0.25 | 17.43 | 4.37 |  |  |  |  |
| Solyc07g041550 | 0.18 | 20.45 | 3.60 |  |  |  |  |
| Solyc03g059420 | 0.20 | 10.47 | 2.13 |  |  |  |  |
| Solyc11g071950 | 0.26 | 18.71 | 4.83 |  |  |  |  |
| Solyc12g099570 | 0.50 | 25.18 | 12.48 |  |  |  |  |
| Solyc10g044900 | 0.50 | 10.90 | 5.50 |  |  |  |  |
| Solyc10g084270 | 0.21 | 13.61 | 2.85 |  |  |  |  |
| Solyc06g016750 | 0.21 | 25.45 | 5.36 |  |  |  |  |
| Solyc02g092380 | 0.19 | 10.74 | 2.03 |  |  |  |  |
| Solyc05g052960 | 0.15 | 18.86 | 2.78 |  |  |  |  |
| Solyc06g009860 | 3.00 | 0.02 | 0.07 |  |  |  |  |
| Solyc10g008950 | 0.22 | 12.91 | 2.80 |  |  |  |  |
| Solyc10g055450 | 0.27 | 25.87 | 7.00 |  |  |  |  |
| Solyc05g006580 | 0.23 | 10.94 | 2.52 |  |  |  |  |
| Solyc03g121310 | 0.20 | 87.27 | 17.67 |  |  |  |  |
| Solyc09g010180 | 0.23 | 39.89 | 9.35 |  |  |  |  |
