# Supplementary material for: Genome-Wide Identification and Evaluation of Reference Genes for Quantitative RT-PCR Analysis during Tomato Fruit Development
Source: Front Plant Sci. 2017 Aug 29;8:1440. doi: 10.3389/fpls.2017.01440 (PMC5581943; doi:10.3389/fpls.2017.01440)
Supplement: Supplementary Table 4 — Ct-values of 38 newly identified RGs during tomato fruit development (IM, MG, B, MR). [file Table4.DOCX]

| Supplemental Table 4. Ct Values of 38 newly identified RGs during tomato fruit development (IM, MG, B, MR) | | | | | | | | | | | | | |
| --- | --- | --- | --- | --- | --- | --- | --- | --- | --- | --- | --- | --- | --- |
|  | IM |  |  | MG |  |  | B |  |  | MR |  |  | Ave |
| *SlFRG01* | 22.206718 | 22.221378 | 22.169146 | 21.318943 | 21.192007 | 21.154627 | 23.646524 | 23.937155 | 23.65803 | 20.302885 | 20.344992 | 20.17407 | 21.86054 |
| *SlFRG02* | 23.334024 | 23.105354 | 23.317476 | 23.300961 | 23.966347 | 23.912069 | 24.287857 | 24.427366 | 23.910444 | 23.029762 | 22.921751 | 23.101116 | 23.551211 |
| *SlFRG03* | 23.950653 | 23.766047 | 23.635239 | 22.931623 | 22.927008 | 22.915987 | 21.476572 | 21.400572 | 21.285509 | 22.742765 | 22.647211 | 22.584723 | 22.688659 |
| *SlFRG04* | 23.938015 | 23.98251 | 24.06617 | 23.134697 | 23.119516 | 23.045597 | 21.899704 | 21.865883 | 21.819632 | 23.210785 | 23.231165 | 23.406218 | 23.059991 |
| *SlFRG05* | 24.044298 | 24.066074 | 23.911173 | 23.239754 | 23.051977 | 23.048502 | 22.950377 | 22.675406 | 22.752878 | 24.478065 | 23.748428 | 24.143244 | 23.509181 |
| *SlFRG06* | 26.766939 | 26.639244 | 26.471012 | 26.909973 | 26.845196 | 26.883818 | 29.972351 | 30.207359 | 30.100613 | 29.592241 | 29.760033 | 29.622906 | 28.314307 |
| *SlFRG07* | 33.075211 | 31.669031 | 33.000233 | 31.422419 | 37.131557 | 33.380714 | 34.383671 | 33.221466 | 34.542229 | 35.239811 | 35.145893 | 34.731319 | 33.911963 |
| *SlFRG08* | 23.010265 | 22.924126 | 22.950649 | 22.934978 | 22.972992 | 22.97193 | 24.019802 | 23.945225 | 23.940071 | 22.13048 | 22.11603 | 22.118233 | 23.002898 |
| *SlFRG09* | 25.842524 | 25.792686 | 25.557638 | 25.98625 | 25.968744 | 25.913893 | 24.920317 | 24.837666 | 24.739647 | 26.568565 | 26.465658 | 27.310688 | 25.825356 |
| *SlFRG10* | 25.9366 | 26.144136 | 25.916058 | 25.402052 | 25.478779 | 25.488529 | 23.509157 | 23.795158 | 23.464607 | 24.223522 | 23.949541 | 23.951794 | 24.771661 |
| *SlFRG11* | 25.33102 | 25.241766 | 25.20295 | 23.966978 | 23.937799 | 23.874964 | 25.225306 | 25.150625 | 25.024761 | 23.70052 | 23.732515 | 23.790312 | 24.51496 |
| *SlFRG12* | 24.221008 | 24.211138 | 24.207863 | 21.856693 | 21.87463 | 21.826307 | 22.04509 | 22.128658 | 22.270489 | 23.214825 | 23.271156 | 23.257877 | 22.865478 |
| *SlFRG13* | 30.786259 | 30.628893 | 30.697144 | 30.788578 | 30.843142 | 30.922159 | 34.929317 | 34.963432 | 34.49564 | 32.706192 | 32.999939 | 32.696819 | 32.288126 |
| *SlFRG14* | 24.860718 | 24.319025 | 24.385899 | 24.783554 | 24.894413 | 24.921934 | 23.644371 | 23.474312 | 23.661934 | 24.718346 | 24.734537 | 24.738012 | 24.428088 |
| *SlFRG15* | 22.75181 | 22.868935 | 22.680347 | 21.177065 | 20.908945 | 20.701073 | 21.125114 | 21.263802 | 21.305138 | 22.929981 | 22.90807 | 22.926378 | 21.962221 |
| *SlFRG16* | 22.748495 | 22.782316 | 22.809914 | 20.740295 | 20.385084 | 20.440554 | 20.908506 | 20.850632 | 20.771717 | 22.033224 | 21.876978 | 21.891285 | 21.519917 |
| *SlFRG17* | 23.972387 | 23.97316 | 23.923365 | 23.807529 | 23.869843 | 23.804789 | 24.284813 | 24.365644 | 24.317818 | 23.347042 | 23.568361 | 23.601278 | 23.903002 |
| *SlFRG18* | 24.444954 | 24.446104 | 24.531631 | 23.547707 | 23.639553 | 23.642334 | 24.727539 | 24.822115 | 25.334146 | 22.429001 | 23.899368 | 23.907324 | 24.114315 |
| *SlFRG19* | Undetermined | 24.868814 | 24.252481 | 22.974323 | 23.344919 | 23.012007 | 25.931917 | 25.490734 | 25.699772 | 26.993496 | 27.052135 | 27.387165 | 25.182524 |
| *SlFRG20* | 19.852381 | 19.920965 | 19.907728 | 18.843191 | 18.882463 | 18.855673 | 20.940157 | 20.893152 | 20.917633 | 20.625156 | 20.444221 | 20.473961 | 20.04639 |
| *SlFRG21* | 22.81105 | 22.689384 | 30.962065 | 21.979084 | 21.944845 | 21.540627 | 21.857544 | 22.230701 | 22.242907 | 22.424631 | 22.397947 | 22.478548 | 22.963278 |
| *SlFRG22* | 24.474773 | 24.396971 | 24.51676 | 24.641582 | 24.575384 | 24.575516 | 25.912083 | 25.858303 | 25.899708 | 26.493319 | 26.518911 | 26.14349 | 25.3339 |
| *SlFRG23* | 27.644369 | 27.588722 | 27.610357 | 26.368023 | 26.322903 | 26.28125 | 24.84897 | 24.840021 | 24.921671 | 26.010611 | 26.10635 | 26.197096 | 26.228362 |
| *SlFRG24* | 27.632231 | 27.791563 | 27.466921 | 25.949129 | 25.892561 | 25.879704 | 24.228298 | 24.293024 | 24.397449 | 25.974703 | 25.887133 | 25.751154 | 25.928656 |
| *SlFRG25* | 21.300364 | 21.229387 | 21.276428 | 19.909548 | 19.870514 | 19.919079 | 20.519861 | 20.499929 | 20.471643 | 20.513449 | 20.702644 | 20.52025 | 20.561091 |
| *SlFRG26* | 26.761005 | 26.75872 | 26.691435 | 24.931465 | 24.918674 | 24.885492 | 23.812967 | 23.843489 | 23.739283 | 25.924122 | 25.773775 | 25.744596 | 25.315419 |
| *SlFRG27* | 25.028505 | 24.92635 | 25.055502 | 24.137182 | 24.156349 | 23.92552 | 22.726442 | 22.762655 | 22.789511 | 23.885338 | 23.98703 | 23.945169 | 23.943796 |
| *SlFRG28* | 25.823931 | 25.911711 | 25.918671 | 25.175425 | 25.178295 | 25.357143 | 25.917871 | 25.911524 | 25.994686 | 27.703636 | 27.718351 | 27.760862 | 26.197676 |
| *SlFRG29* | 24.692598 | 24.73126 | 24.733809 | 23.969835 | 23.981678 | 23.896599 | 24.297121 | 24.268539 | 24.22809 | 25.954226 | 25.970943 | 25.912212 | 24.719743 |
| *SlFRG30* | 22.609676 | 22.245939 | 22.272097 | 21.690163 | 21.642548 | 21.63092 | 19.978556 | 19.953991 | 19.90604 | 20.86342 | 20.88343 | 20.873224 | 21.2125 |
| *SlFRG31* | 26.035807 | 25.956585 | 25.927626 | 24.034502 | 23.99753 | 23.980875 | 23.36306 | 23.304096 | 23.291523 | 25.726072 | 25.883839 | 25.780005 | 24.77346 |
| *SlFRG32* | 25.915775 | 25.723509 | 25.769281 | 24.583359 | 24.543066 | 24.544674 | 23.894201 | 23.848694 | 23.793049 | 26.402531 | 26.208441 | 26.461971 | 25.140713 |
| *SlFRG33* | 28.848879 | 28.899298 | 28.92107 | 26.912525 | 26.926256 | 26.756691 | 26.486053 | 26.590208 | 26.650999 | 29.738815 | 29.910898 | 29.563162 | 28.017071 |
| *SlFRG34* | 29.124847 | 29.164846 | 29.167099 | 29.369207 | 29.316641 | 29.327309 | 28.464127 | 28.586193 | 28.5788 | 30.889797 | 30.704878 | 30.942837 | 29.469715 |
| *SlFRG35* | 23.767725 | 23.754421 | 23.716 | 22.931561 | 23.009817 | 22.95936 | 22.35565 | 22.339546 | 22.606709 | 23.722029 | 23.755753 | 23.783741 | 23.225193 |
| *SlFRG36* | 24.572992 | 24.561012 | 24.718481 | 23.881908 | 23.911474 | 23.940453 | 21.910748 | 21.876884 | 21.891905 | 22.623528 | 22.605944 | 22.648476 | 23.261984 |
| *SlFRG37* | 24.695684 | 24.658604 | 24.772976 | 22.950462 | 22.958183 | 22.930363 | 22.547626 | 22.612419 | 22.562193 | 24.205248 | 24.352388 | 24.250015 | 23.62468 |
| *SlFRG38* | 23.945837 | 23.787416 | 23.870075 | 24.69034 | 24.300627 | 24.255426 | 23.12879 | 23.101007 | 23.904465 | 23.721354 | 23.759462 | 23.760368 | 23.852097 |

IM: Immature fruits; MG: Mature green fruit; B: Breaker fruits; MR: Mature red fruits AVE: Average value
