# Supplementary material for: Genome-Wide Identification and Evaluation of Reference Genes for Quantitative RT-PCR Analysis during Tomato Fruit Development
Source: Front Plant Sci. 2017 Aug 29;8:1440. doi: 10.3389/fpls.2017.01440 (PMC5581943; doi:10.3389/fpls.2017.01440)
Supplement: Supplementary Table 5 — Expression stability (M) values of 38 newly identified RGs evaluated by geNorm and NormFinder. [file Table5.DOCX]

| Supplemental Table 5. Expression stability (M) values of 38 newly identified RGs evaluated by geNorm and NormFinder | | |
| --- | --- | --- |
|  |  |  |
| Gene ID | geNorm | NormFinder |
| *SlFRG01* | 1.229 | 1.163 |
| *SlFRG02* | 1.002 | 0.605 |
| *SlFRG03* | 0.095 | 0.529 |
| *SlFRG04* | 0.156 | 0.423 |
| *SlFRG05* | 0.504 | 0.216 |
| *SlFRG06* | 1.281 | 1.393 |
| *SlFRG07* | 1.147 | 0.857 |
| *SlFRG08* | 1.05 | 0.711 |
| *SlFRG09* | 0.738 | 0.500 |
| *SlFRG10* | 0.675 | 0.724 |
| *SlFRG11* | 0.95 | 0.559 |
| *SlFRG12* | 0.569 | 0.487 |
| *SlFRG13* | 1.343 | 1.609 |
| *SlFRG14* | 0.696 | 0.390 |
| *SlFRG15* | 0.838 | 0.748 |
| *SlFRG16* | 0.547 | 0.454 |
| *SlFRG17* | 0.866 | 0.411 |
| *SlFRG18* | 0.978 | 0.593 |
| *SlFRG19* | 1.189 | 1.165 |
| *SlFRG20* | 1.027 | 0.653 |
| *SlFRG21* | 1.123 | 0.980 |
| *SlFRG22* | 1.074 | 0.753 |
| *SlFRG23* | 0.206 | 0.602 |
| *SlFRG24* | 0.305 | 0.751 |
| *SlFRG25* | 0.715 | 0.177 |
| *SlFRG26* | 1.099 | 0.862 |
| *SlFRG27* | 0.095 | 0.464 |
| *SlFRG28* | 0.922 | 0.681 |
| *SlFRG29* | 0.785 | 0.494 |
| *SlFRG30* | 0.242 | 0.606 |
| *SlFRG31* | 0.594 | 0.675 |
| *SlFRG32* | 0.616 | 0.592 |
| *SlFRG33* | 0.894 | 0.866 |
| *SlFRG34* | 0.81 | 0.631 |
| *SlFRG35* | 0.409 | 0.215 |
| *SlFRG36* | 0.65 | 0.763 |
| *SlFRG37* | 0.467 | 0.452 |
| *SlFRG38* | 0.758 | 0.408 |
